# Workflow analysis of data science code in public GitHub repositories

**DOI:** 10.1007/s10664-022-10229-z

**Published:** 2022-11-19

**Authors:** Dhivyabharathi Ramasamy, Cristina Sarasua, Alberto Bacchelli, Abraham Bernstein

**Affiliations:** Department of Informatics, University of Zurich, Zurich, Switzerland

**Keywords:** Data science, Data science workflow, Data science workflow, Workflow analysis, Data science life cycle, Source code classification, Notebooks, Jupyter notebooks

## Abstract

Despite the ubiquity of data science, we are far from rigorously understanding how coding in data science is performed. Even though the scientific literature has hinted at the iterative and explorative nature of data science coding, we need further empirical evidence to understand this practice and its workflows in detail. Such understanding is critical to recognise the needs of data scientists and, for instance, inform tooling support. To obtain a deeper understanding of the iterative and explorative nature of data science coding, we analysed 470 Jupyter notebooks publicly available in GitHub repositories. We focused on the extent to which data scientists transition between different types of data science activities, or *steps* (such as data preprocessing and modelling), as well as the frequency and co-occurrence of such transitions. For our analysis, we developed a dataset with the help of five data science experts, who manually annotated the data science steps for each code cell within the aforementioned 470 notebooks. Using the first-order Markov chain model, we extracted the transitions and analysed the transition probabilities between the different steps. In addition to providing deeper insights into the implementation practices of data science coding, our results provide evidence that the steps in a data science workflow are indeed iterative and reveal specific patterns. We also evaluated the use of the annotated dataset to train machine-learning classifiers to predict the data science step(s) of a given code cell. We investigate the representativeness of the classification by comparing the workflow analysis applied to (a) the predicted data set and (b) the data set labelled by experts, finding an F1-score of about 71% for the 10-class data science step prediction problem.

## Introduction

Data science (DS) is the process of deriving insights from data (Muller et al. 2019b; Kery et al. 2017; Kim et al. 2016). Due to the continuously growing availability of vast amounts of data and computational capabilities, data science has been adopted across various fields, from drug design to software engineering (Menzies et al. 2016). Being able to properly do data science has become an important skill in today’s world (O’Neil and Schutt 2013; Miller and Hughes D 2017; PriceWaterhouseCoopers 2017). As a result, understanding how to assist data scientists based on their needs is a research area that has gained attention in recent years. While part of the literature focused on conducting interviews and surveys that reveal how data scientists actually work (Wang et al. 2019a; Zhang et al. 2020a; Wang et al. 2021a; Muller et al. 2019b; Wang et al. 2019b; Kery et al. 2018; Kery et al. 2017; Mao et al. 2019; Liu et al. 2020), other works provided technological solutions to improve the way data scientists work in terms of, for instance, collaboration (Patterson et al. 2017), code interaction (Rule et al. 2018; Pimentel et al. 2019; Kery et al. 2019), and automation (Guo and Seltzer 2012; Heffetz et al. 2020; Aggarwal et al. 2019). Our work, instead, provides an empirical investigation of the workflows (i.e., sequence of data science steps) implemented in real-world notebooks that were published on the Web.

A set of studies (Wang et al. 2019a; Zhang et al. 2020a; Aggarwal et al. 2019; Muller et al. 2019b) suggests that a data science workflow is comprised of multiple steps: data acquisition and preparation, preprocessing, feature engineering, modelling, visualisation, and deployment. Low-code data mining systems like RapidMiner (Hofmann and Klinkenberg 2016) and KNIME (Berthold et al. 2009) also group data mining and machine learning procedures and operators in terms of data science steps (data loading and transformation, data preprocessing and visualization, modelling, evaluation, and deployment). A high-level characterisation of such data-oriented steps is provided by Garijo et al. (2013a) through their empirical study of workflows developed in low-code (GUI-based) systems like Wings and Taverna. Such characterisation could help data scientists to reason about and orient themselves in a large collection of data science operators (Garijo et al. 2013a), as well as make sense of existing workflows (Garijo et al. 2013a). However, there is limited work on the empirical characterisation and analysis of workflows developed through code-based workflow development systems like Jupyter. We argue that extending this characterisation of different data science steps for such systems can provide various advantages.

First, it can inform the design of tools and interfaces to support data scientists. Data scientists generally prefer GUIs (Olabarriaga et al. 2014) and need tools similar to an IDE (Integrated Development Environment) (Chattopadhyay et al. 2020). For instance, in traditional IDEs, annotations have proven to be effective in aiding navigation and understandability (Chattopadhyay et al. 2020; Storey et al. 2009). Similarly, data science annotations could provide semantic cues and ease navigation difficulties (Kery et al. 2017) and help users shorten development and debugging time (Olabarriaga et al. 2014). They can help find relevant information (similar to ‘code browser’ in Eclipse) and allow developers to interact with their code easily (McCormick and De Volder 2004).

Second, aiding the analysis of data science workflows can provide insights for workflow development support. Workflow analysis has been well explored in traditional software engineering (SE) (Smith et al. 2005; Meena et al. 2005; Trcka et al. 2008; Colombo et al. 2006; Keith and Vega 2016). Rubin et al. (2007) suggests that improvements to the software process should be aided by the understanding of “what was actually done during the software development process and not by what is simply said about it” (Rubin et al. 2007, p. 170). It seems reasonable that this concept should be adapted to data science workflows as well. Past research studies data-intensive workflows and how they can be supported by low code workflow systems (Garijo et al. 2013a). Using annotations of data science activities in a workflow, these systems are expected to help design tools that allow users to modify, execute, debug, and reuse the (sub-)workflows (Carvalho et al. 2018; Garijo et al. 2013a; Krämer et al. 2013; LaToza and Myers 2010; Carvalho et al. 2017) (e.g., call graph).

Third, better understanding the data science workflows in practice is essential to devise automation and tools that better target data scientists’ needs (Garijo et al. 2013a; Chattopadhyay et al. 2020; Olabarriaga et al. 2014; Storey et al. 2009). For instance, they can provide active support to data scientists by automatically identifying the current step in the workflow under development and recommending the next step and its relevant (alternative) methods. Such identification enables dynamic modelling of both the data science step and its action space compared to the existing systems where the data science step is fixed (Heffetz et al. 2020). Such an AutoML system may incur less computational cost (Heffetz et al. 2020). They can also inform the design of AutoML systems that work as a partner and in collaboration with human practitioners (Wang et al. 2019a; Hernández-Orallo and Vold 2019; Zheng et al. 2017). For example, data scientists work on different steps of the workflow while collaborating in teams (Wang et al. 2019a). Through appropriate interventions (Muir 1994; Norman 1990; Parasuraman et al. 2000) in those steps where humans need help and (or) by automating them, AutoML systems can support a data scientist’s creation and management of data science workflows. Enabling such support requires identification of the steps in a data science workflow (Garijo et al. 2013a; Bennett et al. 2016) and the transitions between them.

In our work, we propose a way to characterise and identify different steps in data science workflows developed through code-based workflow systems like Jupyter notebooks. To that end, we develop an expert-annotated dataset and investigate supervised methods to extend the dataset by classifying the data science code automatically.

Furthermore, while the movement of data through a data science workflow typically hints at a *waterfall*-like pipeline, a few studies suggest that — in practice — data science may be more of an explorative process (Rule et al. 2018) that is iterative (Kery et al. 2019; Rule et al. 2018; Kandel et al. 2012a; Guo and Seltzer 2012) and non-linear (Kery et al. 2017; Rule et al. 2018). These studies provide limited empirical evidence: Rule et al. (2018) use the presence of non-linear execution numbers in a notebook as evidence of non-linearity. However, an execution number may simply reflect code-level changes and not necessarily a step execution. Another empirical study by Garijo et al. (2013a) reports the frequencies of data-related operations in workflows created through low-code workflow systems across domains. However, the study does not discuss the interaction between these operations.

In this paper, we provide further empirical evidence for the non-linearity and elaborate on the interactive patterns in these workflows. To this end, we analyse data science workflows implemented by data scientists to deepen our understanding of the nature of data science coding and the interactions within its steps. Specifically, we investigate which steps are frequently appearing in a data science workflow, whether they co-occur with other step(s), and what kind of interactions happen among them. We provide additional insights into each step of a data science workflow by analysing the number of lines of code and code clones in them. We also aim to further enable a large analysis of workflows in notebooks by creating an expert-annotated dataset of data science workflows and investigating the feasibility of supervised classification methods to expand the dataset.

The objects of our analysis are publicly available data science notebooks implemented in Jupyter. Jupyter is a popular development environment for data science tasks, which allows data scientists to create and share notebooks that contain a sequence of cells. The cells allow the logical separation of code chunks and may include live code, graphical representations of its results, as well as markdown text with equations and other natural language elements like explanations of design decisions, interpretations of results, and links to external documentation. These contextual elements intertwined with the source code contribute to the *computational narrative* (Jupyter 2015) of the notebook.

To conduct our analysis on these data science notebooks, first, we create a novel dataset (DASWOW – **Da** ta **S** cience Notebooks with **Wo** rkflo**w** Information) with the contribution of data scientists. These data scientists manually annotated 470 data science notebooks (selected from the ${\sim }1$M notebooks dataset provided by Rule et al. (2018)), containing 9,678 code cells, by labelling each code cell with the data science step(s) performed within. Second, we use Markov chain modelling to perform a workflow analysis on the dataset to uncover the data science implementation practices. Finally, we evaluate and compare the potential of supervised classification techniques in extending the dataset for large-scale workflow analysis. To train the classifier, we use automatically generated features for each data point (‘code cell’) in the DASWOW along with its corresponding label indicating the data science step it performs.

The main contributions of our study include:

- A *novel, publicly available dataset*, DASWOW, to support workflow analysis and report of a set of descriptive statistics related to code cells, variables, functions lines of code, and lines of comment for the dataset.
- New *empirical insights on data science workflows*. We find that 29.75% of the code cells are written to explore the data — based on the primary purpose of the cell. In line with the common knowledge (Wang et al. 2019a), we also find that 23.5% of the code cells created by data scientists pre-process data. Moreover, 24% of the code cells in Jupyter notebooks implemented *more than one type of data science activity*, thus indicating the (1) overlapping nature of data science activities and (2) data scientists not using Jupyter cell structure to logically separate the data science activities. Our workflow analysis also shows that data science is iterative throughout and at every step in the workflow.
- Evidence that *DASWOW can be extended* with the help of existing supervised classification methods. For this, we employ and evaluate *single-label and multi-label classification methods*; we show that multi-label *random forest classifier* performs better in terms of *F1-score* when trained with features composed of *lines of code and additional software metric information*. We also discuss factors that might impact the performance of the classifier. Furthermore, we repeat the workflow analysis on the predicted dataset and show the representativeness of the classification.

The remainder of this article is organised as follows: Section 2 provides an overview of the relevant related work and lays out the research questions we investigate. Section 3 describes the creation of the expert annotated dataset — DASWOW. Section 4 presents the workflow analysis on DASWOW and details the insights we obtained. Section 5 shows the potential of supervised classification methods in extending the dataset and further discusses the evaluation results. We also report the workflow analysis on the predicted dataset and showcase its representativeness. Section 8 concludes the article with a brief summary of the insights and a discussion on the results’ potential.

## Background and Research Questions

We provide an overview of the related work that leads to the main research questions (RQs) addressed in this article. Specifically, we look at the literature on data science workflows and their classification.

### Characterisation of Data Science Workflows

A data science workflow consists of data-related activities that range from obtaining the data to modelling or interpreting it. Different studies consider that a data science workflow includes (some of) the following steps: data acquisition and preparation, preprocessing, feature engineering, modelling, visualisation, and deployment (Wang et al. 2019a; Zhang et al. 2020a; Aggarwal et al. 2019; Muller et al. 2019b; Souza et al. 2020). Garijo et al. (2013a) identified a similar set of data-related activities in the case of scientific workflows. In addition to the above, grey literature also includes other activities like business understanding/problem framing, data exploration, and reporting (Springboard 2016; Microsoft 2020; UCSD 2021). Other studies group the list of data science steps into a set of high-level tasks. For example, Kandel et al. (2012b) suggests the existence of the following tasks: discovery, wrangling, profiling, modelling, and reporting, whereas Kun et al. (2018) proposes questions, data, methods, inference, and design.

Given that computational notebooks are popular tools for data science implementations, a large-scale analysis of how these data science steps are implemented in existing notebooks can support understanding the data science workflows in practice. This requires a data set that contains expert annotations of data science workflows in data science notebooks. Zhang et al. (2020b) produced a dataset of 100 notebooks (randomly selected from Rule et al. (2018)) by assigning a label from a set of high-level data analysis task categorisations — ‘IMPORT, WRANGLE, EXPLORE, MODEL, EVALUATE’ — per cell.

However, the existing task categorisations do not represent the data science workflows in notebooks exhaustively. For example, some code fragments are also used to support the analysis in the form of helper functions (e.g., import pandas), to aid data loading, and visualisation. Many notebooks also have code fragments that support saving the obtained results. Therefore, to facilitate an empirical investigation of data science workflows in notebooks, this paper identifies the relevant steps in a data science workflow from the literature and extends them to computational notebook platforms. We provide annotations that contain the steps mentioned above and include the steps that are identified through literature in order to provide a complete characterisation of data science workflows in notebooks. We then use these steps as labels to manually annotate the notebook cells resulting in our first contribution: the **DASWOW** dataset. As we will outline in Sections 3 and 4, this approach allows us to better understand the inner workings of data science analyses.

### Analysis of Data Science Workflows

The analysis of software development workflows and processes has been explored in the field of SE by several studies (Smith et al. 2005; Meena et al. 2005; Trcka et al. 2008; Colombo et al. 2006; Keith and Vega 2016; Rubin et al. 2007; Chan and Leung 1997).

Researchers, particularly from HCI and CSCW community, have explored data science practices (Zhang et al. 2020a; Kross and Guo 2019; Mao et al. 2019; Muller et al. 2019b; Passi and Jackson 2018; Rule et al. 2018; Wang et al. 2021a) and how data scientists work with computational notebooks like Jupyter (Kery et al. 2019; Muller et al. 2019b; Kery et al. 2018; Kery et al. 2017; Rule et al. 2018; Chattopadhyay et al. 2020), primarily through surveys or expert interviews of data scientists. These studies are focused on understanding data science practices and workflows with a human-centric approach (Aragon et al. 2016; Muller et al. 2019a). Particularly, Kross and Guo (2019) found that instructors of data science emphasised the creation of computational workflows that center on ‘code, data, and communication’ during their teaching. Whereas, Zhang et al. (2020a) studied how data science teams collaborate through several stages of a data science workflow. In this work, we perform a quantitative study of workflows by characterising and empirically analysing the resulting artefacts of a data science process i.e., computational notebooks. As a result, we view our work to be complementary in enhancing our understanding of data science workflows in practice.

Previous research on scientific workflow analysis (Garijo et al. 2013a; Garijo et al. 2013b; Liu et al. 2015) focuses on workflows developed through low code systems like Taverna (Missier et al. 2010) and Wings (Gil et al. 2010) that allow the creation of scientific workflows through drag and drop interface and involve less or no coding. Studies on such workflows are concerned with issues like the management of these scientific workflows to provenance tracking in order to create reproducible and reusable workflows. One such study by Garijo et al. (2013a) focused on abstracting the data-intensive scientific workflows to identify the different types of tasks carried out in the workflows. Such abstractions can facilitate the understandability and reuse of workflows (Garijo et al. 2013a). In our work, we adapt and extend such characterisation for code-based computational notebook systems like Jupyter, further analysing the sequence in which data science steps (co-)occur.

There is currently no work^1^ that investigates the workflows developed through computational notebooks in order to understand the steps implemented in them, the details of their iterative nature and behaviour (interactions). While the literature documents the iterative and non-linear nature (Kery et al. 2019; Kandel et al. 2012a; Guo and Seltzer 2012; Kery et al. 2017; Rule et al. 2018) of data science notebooks, there is, yet, little empirical work on the analysis of workflow in data science notebooks. Rule et al. (2018) empirically analysed the iterative nature of Jupyter notebooks using the execution number available for each of the cells. Other studies focused on specific steps in the data science workflow (Lee et al. 2020; Hohman et al. 2020). Zhang et al. (2020b) analysed the role of individual steps in scientific data science workflows in academic publishing and how they differ differences between scientific domains. Lee et al. (2020) analysed workflows shared in the OpenML platform to extract information on the usage of machine learning (ML) techniques.

In our study, we focus on identifying whether some steps are more frequent in the workflow than others, with which other step(s) they frequently co-occur (if any), and how the steps interact with each other throughout the notebook. We also look at the distribution of lines of code across the workflow. Finally, given that anti-patterns such as code clones (Brown et al. 1998) are prevalent in computational notebooks (Koenzen et al. 2020; Källén and Wrigstad 2020) and are often investigated in software engineering analysis (Rahman et al. 2012; Roy and Cordy 2007), we also investigate in what data science steps clones occur in a data science workflow. Specifically, we answer the following questions: 
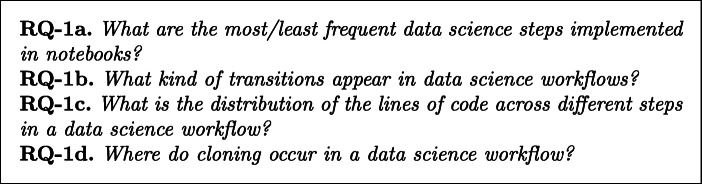


Previous studies analysing software workflows have used several analysis techniques including (timed) Markov-models (Smith et al. 2005; Colombo et al. 2006), activity diagrams (Meena et al. 2005) and Petri-nets (Trcka et al. 2008; Rubin et al. 2007). As we model the data science steps as states, we use the Markov chain model in order to compute the transition probabilities between them, assuming that the prediction of the next step is influenced only by the current state.

### Classification of Source Code

Methods for classifying source code for various purposes (e.g., identification of programming language, authorship, topic, and quality) have been studied extensively in the field of software engineering. Particularly, machine learning techniques have been evaluated for their performance in classifying source code. Several techniques like Max-Entropy Classifier (ME) (Zevin and Holzem 2017), Support Vector Machines (SVM) (Ugurel et al. 2002), Decision Trees (DT) (Knab et al. 2006; Barstad et al. 2014), K-Nearest Neighbour (KNN) (Barstad et al. 2014), and Naive Bayes (NB) (Barstad et al. 2014) have been explored for source code classification. Applications of these techniques include prediction of programming language (Zevin and Holzem 2017; Ugurel et al. 2002), documents’ topic or content (Ugurel et al. 2002; Bacchelli et al. 2012), defects (Knab et al. 2006; Pascarella et al. 2019), code comments (Pascarella and Bacchelli 2017), code authorship (Pellin 2000), and code quality (Barstad et al. 2014).

Several features including code metrics (Barstad et al. 2014), (lexical) tokens — code, header file names, comments, README etc. represented in terms of *n-grams* and/or *phrases* (Zevin and Holzem 2017; Ugurel et al. 2002), syntactical structures (Zevin and Holzem 2017; Pellin 2000), and coding style (Pellin 2000) have been investigated in source code classification problems. Bacchelli et al. (2012) use sequence capturing features to classify e-mail messages related to software development using features such as @@-lineBefore and @@-lineAfter to consider the preceding and subsequent lines of code to recognise the structure of patch or stack trace content.

In terms of research on the classification of data science steps in source code, specifically, based on the workflow information, Zhang et al. (2020b) proposed weakly supervised transformer-based architecture to classify and assign source code to high-level data analysis tasks. In this article, we present our implementation and evaluation of a supervised data science code classification method by formulating it as a *topic* classification problem. We also investigate two classification strategies: single-label (one label per cell) and multi-label classification strategy to account for the potential existence of multiple data science activities in one given cell. Furthermore, we use the insights from existing SE literature to guide our feature and model selection for the classification. We structure our research to answer the following research questions: 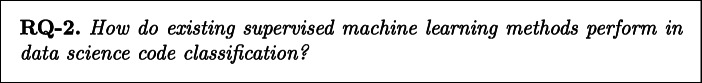

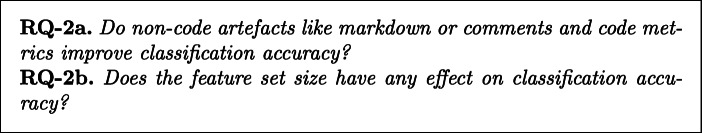


## DASWOW: a Labelled Data set of Data Science Notebooks with Workflow Information

As previously discussed (Section 2), there is currently no annotated dataset to conduct an analysis of workflows in data science notebooks. Therefore, to conduct our investigation, we created DASWOW, a dataset of publicly available data science notebooks from GitHub that are annotated with workflow information. To allow other researchers to continue in this line of work, we make DASWOW publicly available together with the analyses here: 10.5281/zenodo.5635475. In the rest of this section, we detail how we created this dataset.

### Notebook Selection Procedure

We based DASWOW on a corpus of about one million Jupyter notebooks retrieved from GitHub by the Design Lab team at UC San Diego, which contains both the repository contents (including the code and other associated files such as README.md files) as well as repository metadata information such as owner information (Rule et al. 2018). Python notebooks account for a large portion of the corpus (96.04%), although it also contains notebooks in Julia (1.19%) and R (1.16%). The notebooks are obtained from over 1,000 repositories that were generated for diverse purposes (e.g., data science tasks, homework submissions, and software development (Rule et al. 2018)).

As we focus on data science notebooks, we manually inspected several notebooks to select a subset of 500 notebooks based on the following conditions:

*Purpose:* The notebook was developed for a data science task^2^ and has at least a cell.
*Kernel language:* The notebook uses *Python* as their kernel language.

Among the different types of cells supported in Jupyter notebooks (i.e., Markdown, Code, Raw NBConvert, and Output), we focus on code cells (i.e., we regard each code cell as a data point) as they provide a separation of the code snippets. Also, to implement a particular data science step, usually more than a single line of code is needed. Code cells contain lines of code written in the language associated with the kernel (Python in our case) and, on execution, produce a list of outputs that are displayed in the Output cell. Code cells can also contain code comments and may be denoted with an execution_count which indicates the order in which it was executed.

### Annotation Labels

As the first step to the annotation task, we identify the existing data science activities by looking at the literature and online resources that discuss data science practices and extend them to computational notebooks. As we only focus on programming activities, we ignore steps that occur outside of notebooks, such as understanding and framing the problem, data collection, and ETL (Extract-Transform-Load), which is done with specialised tools (Vassiliadis et al. 2009), and deployment (Kery et al. 2018). As a result, we identify the following steps as part of data science workflows in notebooks: data_exploration, data_preprocessing, modelling, evaluation, as well as prediction. In addition, we consider loading the data into the notebooks as a different step (load_data) in the workflow as we noticed that these steps are generally performed in separate cells. Similarly, cells that visualise the results (result_visualization) and save them (save_results) for later use are found in Jupyter notebooks. We also identify cells that are being used for importing libraries, setting environmental parameters etc., which usually happens at the beginning of the notebook — as helper_functions. Additionally, we also encountered code cells that contain only comments (either code comments or commented code denoted as comment_only). As a result of this process, we propose the ten labels as listed in Table 1 to represent the steps in a data science workflow in Jupyter notebooks and use them for our annotation task. We position the proposed labels for computational notebooks motivated by (prominent) existing categorisations in the literature in Table 2.

**Table 1:** Data Science steps annotated in DASWOW

Label	Definition
helper_functions	Code that is not directly related to the data science activity at hand, but provides useful scripting functions (e. g. importing or configuring libraries).
load_data	The process of loading a dataset of any type (e.g., .csv, .pkl) into a Jupyter notebook environment.
data_preprocessing	The process of preparing the dataset(s) for the subsequent analysis. It includes tasks such as cleaning, instance selection, normalisation, data transformation, and feature selection.
data_exploration	The process of inspecting the content and shape of a dataset to understand the nature and characteristics of the data. Note that it may involve the usage of visualisation techniques but differs in its purpose.
modelling	The process of applying statistical models and learning-based algorithms to learn from sample data.
evaluation	The process of assessing a model using one/various evaluation metric(s) such as goodness of fit and accuracy.
prediction	The process of applying a model trained on a set of data to other or newly arriving pieces of data to forecast new values.
result_visualization	The process of obtaining a graphical representation (e.g., tables, plots, graphs) of a/several measurement(s)
save_results	The process of serialising and storing the data.
comment_only	Lines of comment including commented code.

**Table 2:**
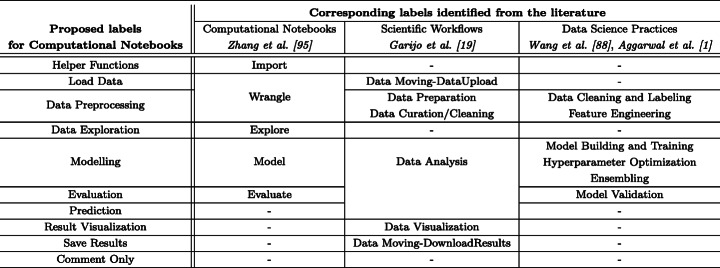
We showcase the proposed labels against categorisations from three different lines of work: computational notebooks, scientific workflows, and data science practices

We consider those labels that correspond to coding activities. Steps taken before the data is loaded into the notebooks (e.g., Data Acquisition) and steps that may occur outside notebooks (e.g., Deployment) are not included

During the annotation activity, each code cell in the notebook is assigned a *primary label* — the main activity performed in the cell — along with other relevant labels that we define as *secondary labels* based on their presence. For example, if a cell performs modelling activity but also visualises the generated model within the same cell, then, we identify modelling as the *primary label* and result_visualization as a secondary label. The detailed set of instructions used for the annotation task is provided in the Appendix (Section Appendix).

### Expert DASWOW Annotation

Five data science experts^3^ (E1^*^, E2^#^, E3^#^, E4^‡^, E5^‡^)^4^ annotated the selected 500 notebooks according to the following procedure. We conducted two pilot studies: one, to improve the annotation task setup and two, to hire external annotators with data science expertise in order to ensure consistent and quality labelling during the main annotation activity.

First, we conducted a pilot study with three experts (E1, E2, E3) who annotated a preliminary set of 50 notebooks based on an initial set of instructions in order to assess the clarity of the instructions and quality of the annotations. We used Cohen’s kappa (Cohen 1960) to assess the inter-annotator agreement between the expert’s annotations (all the relevant labels including *primary label*) for the 50 notebooks. Table 3 lists Cohen’s kappa for the different labels (i.e., data science steps), comparing the performance of E1 with E2 and E3. Based on the interpretation by Altman (1990), most of the agreements are between ‘moderate’ and ‘good’. Annotators saw data_preprocessing in some instances as an extra step and thus, wrongly ignored it during the labelling leading to a ‘low’ agreement. Similarly, save_results had only a ‘fair’ agreement (highlighted rows in the table) as it occurred along with other steps and was seen as incidental rather than as an activity on its own. Another disagreement was that data visualisations that were a result of exploration activity were sometimes classified as result_visualization while we expected annotators to classify them as data_exploration according to the instructions. A different case of disagreement arose when annotators did not distinguish specific labels and considered them sub-set of other labels despite the instructions. For example, some annotators labelled evaluation and prediction actions as modelling even when corresponding distinct labels were available. In most cases, the disagreement was primarily driven by a misunderstanding or a subjective understanding of the definition, misreading the purpose of the code^5^, and human error. After holding discussions among E1, E2, and E3, we resolved the disagreements by i) annotating a visualisation as data_exploration when it is a result of exploration activity, ii) emphasising model evaluation and model prediction activities with their corresponding labels, and iii) clarifying the details explicitly by revising the instructions with examples.

**Table 3:**
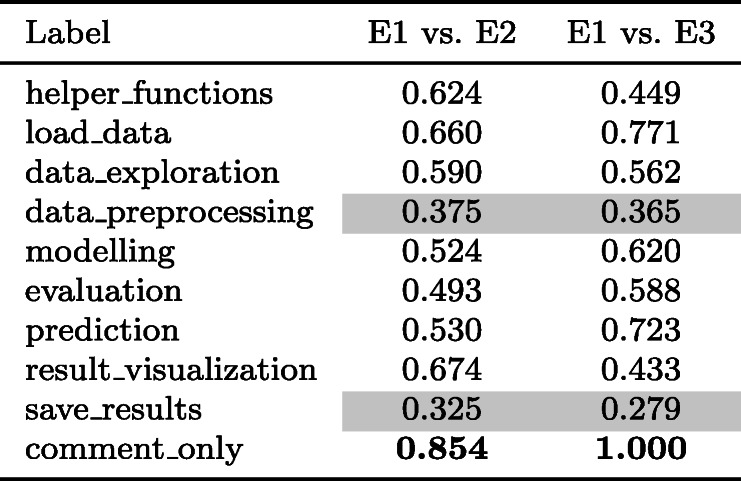
Annotator agreements from the first pilot task

Cohens kappa score for the DS step annotation task. (0-0.2: Poor, 0.2-0.4: Fair, 0.4-0.6: Moderate, 0.6-0.8: Good, **0.8-1**: Very good). Bold numbers in the table indicate the top score, and numbers in coloured background cells indicate poor/fair scorers

As a result of the preliminary annotation task, we improved the instructions (available in Section Appendix). We also incorporated the feedback from the experts to revise the annotations of the preliminary set of 50 notebooks (done by E1). We conducted a second pilot study in order to select further annotators for the annotation task, for which we invited five Upwork freelance data scientists selected based on their experience with data science tasks. They were asked to annotate 30 randomly selected notebooks of the preliminary set. We manually checked the quality of these annotations and also used Cohen’s kappa measure (refer to Table 4) to evaluate the annotations of each of the participants against E1’s annotations (which were updated based on the discussion with E2 and E3 as described above). Based on the results, we hired two of the five experts (E4 and E5) for the rest of the annotation process.

**Table 4:**
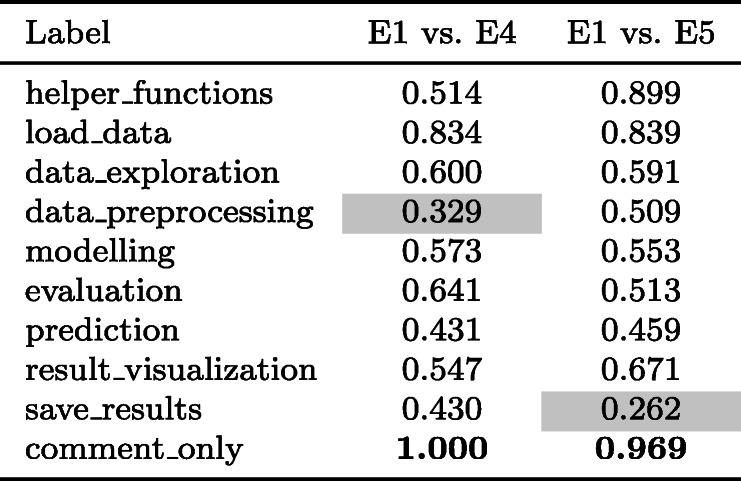
Annotator agreements from the second pilot task

Cohen’s kappa score for the DS step annotation task. (0-0.2: Poor, 0.2-0.4: Fair, 0.4-0.6: Moderate, 0.6-0.8: Good, **0.8-1**: Very good). Bold numbers in the table indicate the top score, and numbers in coloured background cells indicate poor/fair scorers

As a next step, E1 separately annotated further 50 notebooks. E4 and E5 annotated 200 notebooks each independently, taking the total to 500 notebooks (together with the preliminary set of 50 notebooks). As a result, each of the 500 notebooks is annotated by one of the experts. From the 500 notebooks we labelled in total, we retained notebooks with at least one of the following data science types of actions: data pre-processing, data exploration, modelling, and result visualisation. At the end of this process, we were left with 470 data science Python notebooks that we considered our object data for further analysis.

### DASWOW Descriptive Statistics

**Fig. 1:**
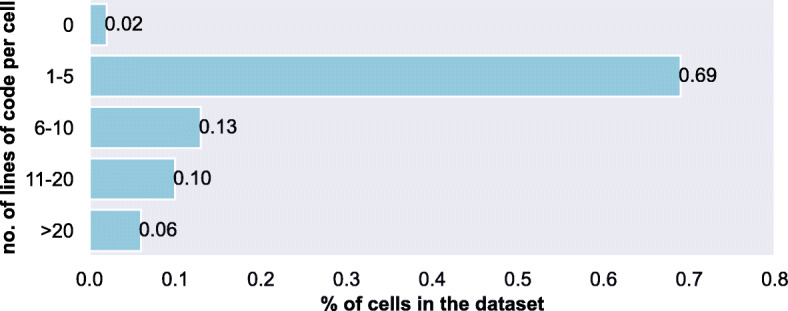
Number of lines of code per cell

DASWOW contains data on 470 notebooks containing 9,678 code cells with a total of 59,128 lines of code and 9,004 lines of comments. Figure 1 shows the distribution of the number of lines of code per cell in the dataset. The majority of the cells (${\sim }70$%) have 1 − 5 lines of code. Table 5 provides further descriptive statistics for DASWOW. On average, a notebook has around 20 code cells and 125 lines of code; lines of comment are comparatively fewer (*mean* = 19), which is in line with observations from other studies (Rule et al. 2018). The statistics also reveal a substantial use of variables, whereas functions are scarcely used.

**Table 5:** DASWOW: Notebook-level statistics for 470 notebooks

	no. of	no. of	no. of	no. of	no. of
	code cells	variables	functions	lines of comment	lines of code
mean	20.591	64.936	3.034	19.157	125.804
std	18.458	77.246	5.675	37.004	138.732
min	1	1	0	0	4
25%	9	23	0	2	49
50%	15.5	42.5	1	10	82
75%	26	74	4	23	142.75
max	162	732	46	523	1052

## Workflow Analysis of Data Science Code

One of the central questions when investigating how to best support data scientists in their analysis is to understand the progression of the analysis that they go through. That is, we need to better understand the progression of higher-level activities — the data science steps — and their inter-relationships. As a result, the remainder of this section is organised around a workflow analysis addressing research questions (Section 2.2) referring to the *frequency* in which the different types of data science steps occur across notebooks, the way these types of steps *co-occur* in code cells, and *transitions* between them.

### **RQ-1a.***What are the Most/Least Frequent Data Science Steps Implemented in Notebooks?*

*Methodology:* We compute the frequency distribution of the annotation labels that indicate the data science step(s) in each code cell of the Jupyter notebooks in DASWOW.

**Fig. 2:**
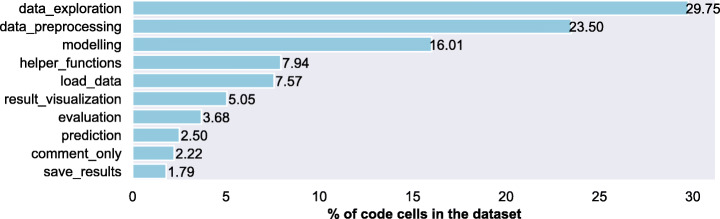
Percentage of cells per label: Primary label

*Results:* When we consider that cells have only *primary label* (assuming cells have a main purpose i.e., to perform a single data science step), we find that, around 23.5% of the cells perform data_preprocessing and around 30% of the cells perform data_exploration (Fig. 2 shows the composition of all the data science activities in the DASWOW).

**Fig. 3:**
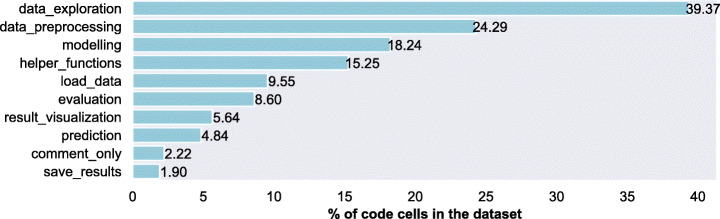
Percentage of cells per label: All labels

When we acknowledge that cells can refer to multiple data science steps, having a primary and a secondary step/label, the distribution slightly changes, as shown in Fig. 3. In the multi-label case, the order of evaluation (6.6%) and result_visualization (4.3%) is flipped compared to the single label case (with 3.68% and 5.05% respectively). This increased occurrence of result_visualization as a *secondary label* could indicate that, after a certain activity, results are usually visualised. Apart from this, we notice that helper_functions is one of the most frequently appearing labels, which indicates data scientists import many external libraries into their notebooks.

We also distinguish between notebooks that contain a modelling label and notebooks that do not contain a modelling label to understand the composition of data science steps in modelling tasks. Particularly, we want to check whether data_exploration is still prominent in modelling tasks. We compute their distribution of labels to understand the frequency of data science activities when a certain type of data science task (i.e., modelling or exploration) is performed.

**Fig. 4:**
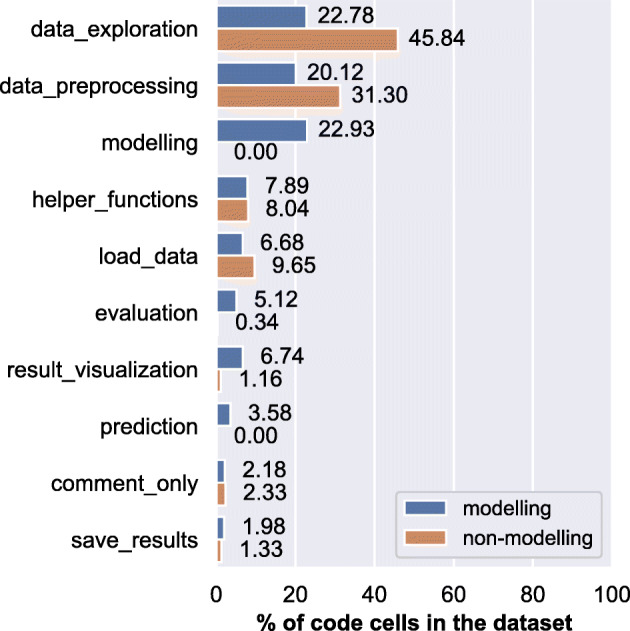
Percentage of cells per label by type of data science task: All labels. Modelling tasks perform data modelling and, in many cases, focused on predicting future outcomes. Non-modelling tasks do not model data and are instead focused on processing, exploring, or analysing the data

The results (refer to Fig. 4) show that when notebooks include the modelling step, data_exploration and data_preprocessing account for 42.9% of the labels. In the case of notebooks that do not include the modelling step, data_exploration and data_preprocessing account for 77.14% of the total labels. The results show that result_visualization is more frequent in notebooks that include modelling. As expected, evaluation and prediction appear only in those notebooks that contain modelling step. The interesting finding is that data_exploration is consistently the most frequent data science step in notebooks whereas according to the literature, data_preprocessing usually takes the majority of time (Muller et al. 2019b; Zhang et al. 2020a).

Furthermore, around 76% of the cells in our data set have one data science step, 19% have two steps, and 4.85% have more than two steps. In order to find out the frequently co-occurring labels within a single cell, we compute the frequency distribution of the label sets (all the labels per cell) in the dataset as the co-occurrence of certain data science steps or label sets (i.e., the presence of a certain combination of labels) could indicate a logical grouping of activities in the data science workflow. We observe co-occurrence for each possible label set. Table 6 shows the top-3 most frequently co-occurring label steps of sizes 1,2,3, and 4, i.e., labels that appear together in a cell but the cell may contain other labels as well. Most often (5.25%) co-occurring 2-label set is (data_exploration, data_preprocessing). This could indicate that exploration and pre-processing of data are activities that often occur in conjunction. Other frequently co-occurring data science label sets (excluding the general labels) with a share of at least 1*%* are (evaluation, modelling) at 4.27%, (modelling, prediction) at 1.95%, (evaluation, prediction) at 1.94%, (data_exploration, modelling) at 1.46%, and (evaluation, modelling, and prediction) at 1.16%. This indicates a logical grouping of activities in the data science workflow as expected, for example, similar to (Wang et al. 2019a).

**Table 6:** Top-3 frequently appearing label combinations: a set of 1-label, 2-label, 3-label, and 4-label in the dataset

Combination of Labels	% of co-occurrence	% appearance as a set
1-label		
(data_exploration)	39.37%	27.76%
(data_preprocessing)	24.29%	16.74%
(modelling)	18.24%	9.53%
2-label		
(data_exploration, data_preprocessing)	5.25%	4.59%
(evaluation, modelling)	4.27%	2.18%
(data_exploration, load_data)	3.3%	2.39%
3-label		
(evaluation, modelling, prediction)	1.16%	0.53%
(evaluation, helper_functions, modelling)	0.85%	0.42%
(data_exploration, helper_functions, load_data)	0.63%	0.52%
4-label		
(evaluation, helper_functions, modelling, prediction)	0.32%	0.27%
(evaluation, modelling, prediction, result_visualization)	0.17%	0.13%
(evaluation, helper_functions, load_data, modelling)	0.11%	0.08%

*% of co-occurrence* indicates how frequent the combination of labels appears (may have other labels in the same cell) in the dataset. *% appearance as a set* indicates how frequent the combination of labels appears as a set (without other labels in the cell) in the dataset

We also obtain the top-3 most frequent label sets of sizes 1, 2, 3, and 4 in DASWOW, i.e, labels that appear as a set in a cell without other labels. data_exploration and data_preprocessing appear often (4.59%) as a label set. Other frequent data science label sets are (evaluation, modelling) at 2.18% and (data_exploration load_data) at 2.39%.

We suspect another possible reason for the co-occurrence of data science steps is the lack of structure (Rule et al. 2018) and modularity (Desmond 2020) of data science code — resulting in large code chunks mixing various types of activities that are conducted in the same cell.

### RQ-1b. What kind of Transitions Appear in Data Science Workflows?

*Methodology:* We compute the transition probabilities between types of steps using a first-order Markov chain model, where states represent the types of data science steps.

For each notebook, we set a start state and a stop state at the beginning and end of the notebook, respectively. Thus, the first cell has an incoming transition from start and the last cell in the notebook has an outgoing transition to stop. For example, a notebook with three cells will be mapped to: 
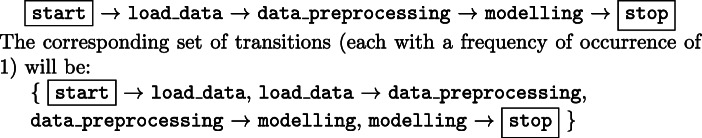


Once the transitions are identified, we compute the transition probability matrix. For cells that have been labelled with multiple labels (i.e., containing secondary labels), as we lack the information about the order of their appearance, we “naively” assign each edge in the transition equal probability using the formula: 1/*no*_*of*_*edges*.

**Fig. 5:**
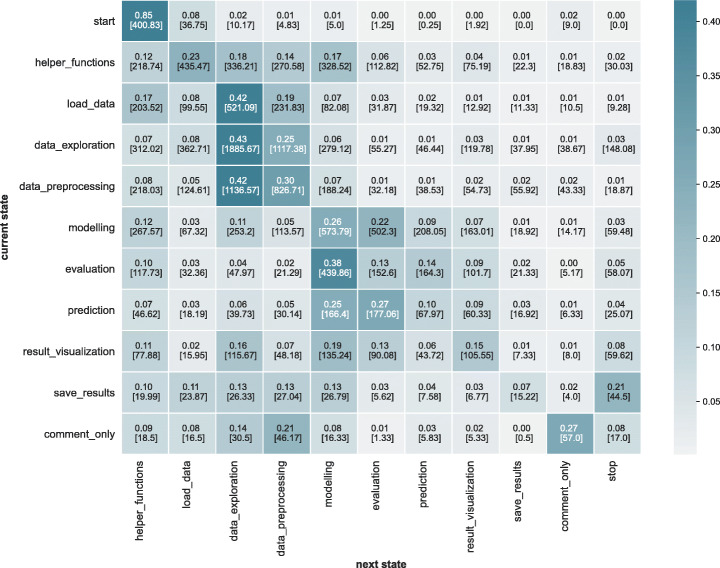
Transition matrix probabilities for the data science workflows. In each cell, the number on the top indicates the % of transitions from the current state to the next state, and the number on the bottom indicates the absolute frequency of transitions

**Fig. 6:**
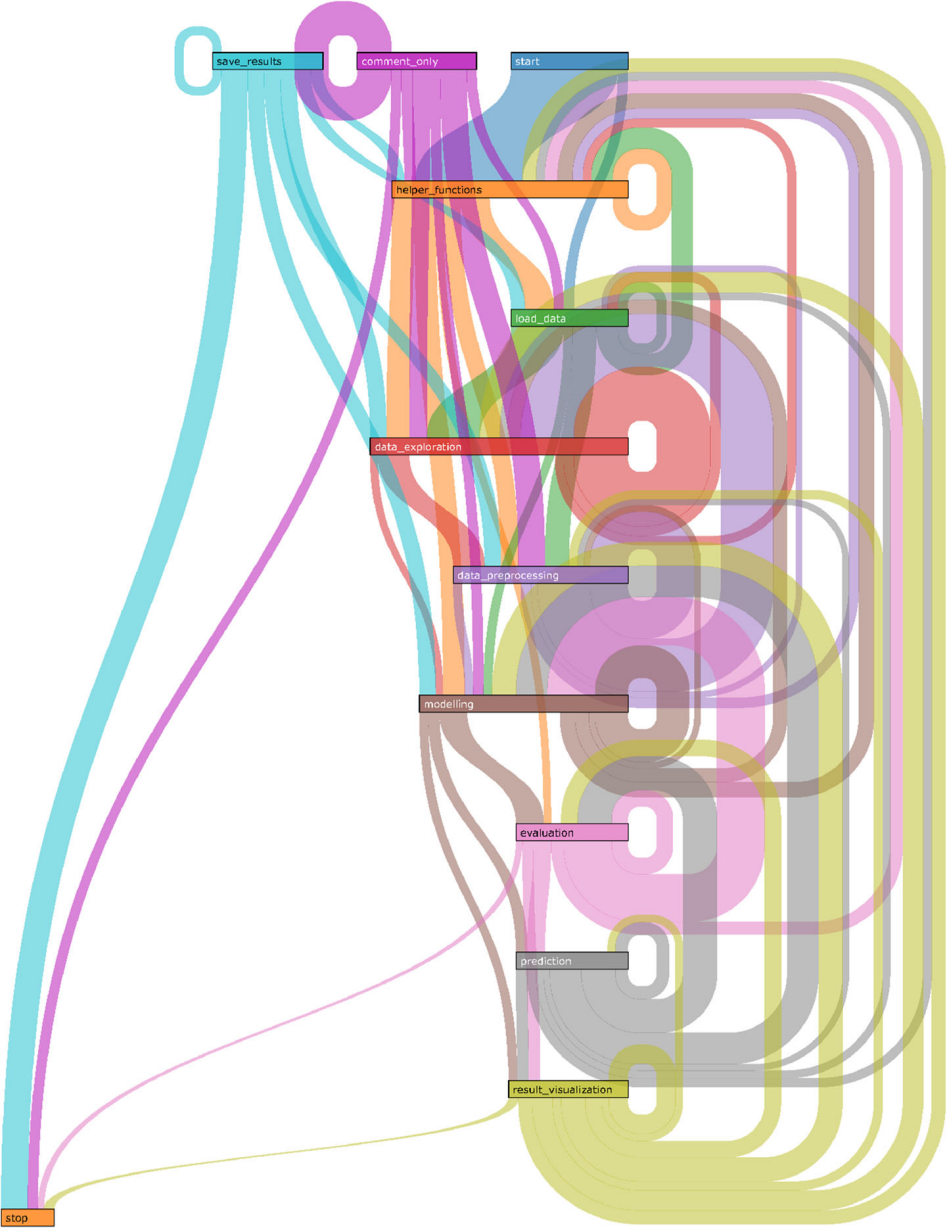
Transition matrix probabilities (> 0.05) visualised in a Sankey diagram. While modelling, evaluation and prediction is iterative as expected, the workflow throughout its life cycle shows iterative-ness

*Results:* Figure 5 visualises the resulting transition probability matrix which reveals unique patterns of prominent iterations. Figure 6 visualises the transition matrix as a Sankey Flow Diagram (showing only transitions with probability > 0.05 for readability). The diagram shows that the data science workflows are iterative, not only at certain steps (e.g., modelling) but throughout its life-cycle. It reveals unique iterative patterns among and within different types of data science steps missing in the previous works, particularly within the following set of steps: (data_preprocessing, data_exploration), and (modelling, evaluation, prediction). More than 25*%* of the transitions starting at data_exploration flows into data_preprocessing and vice versa. Similarly, 22*%* of the transitions flow from modelling to evaluation, 38*%* from evaluation to modelling, 25*%* from prediction to modelling, and 27*%* from prediction to evaluation.

Furthermore, the matrix reveals that not only are there transitions between types of data science steps but also within types of steps (i.e., there are loops around the same step). The steps data_exploration, data_preprocessing, and modelling show that more than > 25*%* of the iterations take place within the same step type.

### RQ-1c. What is the Distribution of the Lines of Code Across Different Steps in a Data Science Workflow?

*Methodology:* We compute the number of lines of code for each of the data science steps as *primary label* (per cell) in DASWOW.

**Fig. 7:**
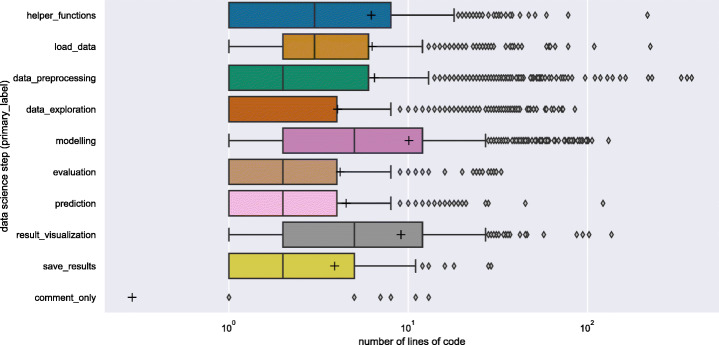
Distribution of the number of lines of code (in log scale) per label considering the primary label. ‘+’ marker indicates the mean value. The mean values for all the labels are higher than the median and are in the upper quartiles

*Results:* Figure 7 shows the results using a box plot. To draw our inference, we assume that number of lines of code can refer to the potential effort a data scientist must invest in implementing a particular data science step. We consider the assumption reasonable even when some of the lines of code are clones, given one should put effort into modifying and(or) understanding the code clone to the task in hand (Kery et al. 2017). We find that, in general, modelling takes considerable effort as it contains a large number of lines of code. Although, if we consider the outliers, data_preprocessing step has the highest number of lines of code written.

If we ignore the outliers, our dataset shows that the initial steps of load_data, data_preprocessing, and data_exploration together account for a similar amount of code written for modelling step. Furthermore, a large number of lines of code are written for result_visualization showing that data scientists consider the communication of results as an important activity. Apart from this, we notice that a large number of lines of code are written for helper_functions which reiterates the finding (refer to Section 4.1) that data scientists import many external libraries into their notebooks.

### RQ-1d. Where do Cloning Occur in a Data Science Workflow?

*Methodology:* We compute the number of lines of code that is *identically* (i.e., type-1) cloned, appearing at least three or more times (by following the ‘rule-of-three’ (Neill et al. 2011)) within the same notebook in DASWOW. In order to understand the purpose of the code that is being cloned, we use the *primary label* of the cell where every time the code appears. We use the number of appearances and *primary label* in conjunction, i.e., a clone appearing at least three times in the cell(s) with the same *primary label*. It is possible some of the clones may appear in cells that have a different primary purpose. We leave their detailed study and study on other types of clones^6^ to future work.

**Fig. 8:**
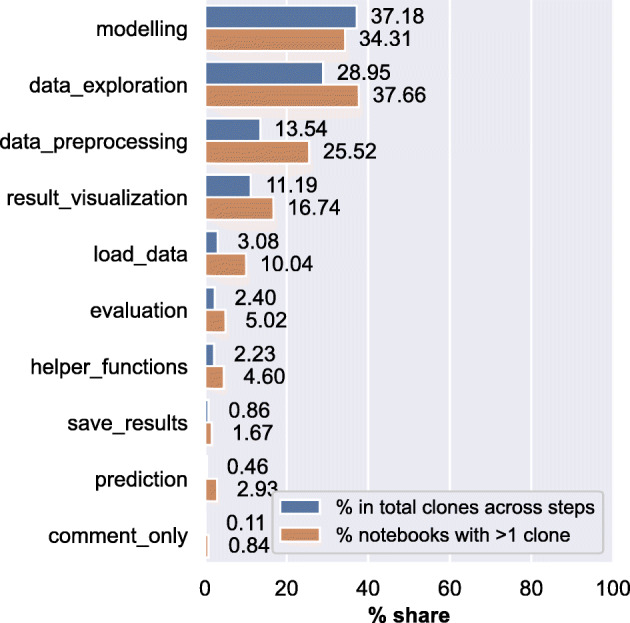
Of the notebooks that contained clones, the plot shows the percentage share of type-1 clones is shown alongside the percentage of notebooks with a given type of clone

*Results:* We find in our dataset that ${\sim }51\%$ of the notebooks have at least one line of code cloned in them. Of the notebooks that have clones, eight (or more) lines of code are cloned on average per notebook, and ${\sim }26\%$ of the notebooks have clones higher than average. In Fig. 8, we show the percentage share of type-1 clones in DASWOW based on what data science step they appear in alongside the percentage share of notebooks that have clones in that data science step. Our results from data science notebooks are in line with the trend of a high occurrence of clones in Jupyter notebooks in general (Koenzen et al. 2020; Källén and Wrigstad 2020). Additionally, DASWOW allows us to understand the clones at a spatial level by situating them in the data science workflow.

We find that most of the type-1 clones (${\sim }66\%$) appear in modelling and data_exploration step. A large number of clones in these steps could mean data scientists repeating or iterating over these steps, for example, executing a scikit model again after modifying the data. Our results also show that modelling and data_exploration are also the most likely steps to be cloned in a notebook, as they appear in > 30*%* of the notebooks in DASWOW.

Besides, our analysis shows a conservative account of clones in data science notebooks. We hypothesise that relaxing our conditions of *identical-ness*, a minimum number of appearances (three or more) in the same step, and within-notebook duplication could lead to a higher number of clones appearing in each step (e.g., import statements, scikit functions). Whilst code clones are usually seen as a bad practice in SE (Fowler 2018) for creating overhead in maintainability and comprehensibility (Roy and Cordy 2007), there might be reasons why they may be acceptable or inevitable (Källén and Wrigstad 2020) in data science notebooks, for example, to simplify development effort (Kery et al. 2018; Koenzen et al. 2020). Hence, future work will have to investigate how to support and handle code clones, particularly in steps where they appear frequently.

### Summary

Our workflow analysis provides insights that can support designing tools that assist data scientists while creating and managing their data science workflows. We discuss the implications of the findings in detail in Section 6. In order to further cater to various needs, a large-scale workflow analysis on data science notebooks based on different dimensions (like task type and domain) could provide further helpful insights for designing tools that support data science development. However, the process of manually labelling a dataset is expensive. Hence, in the next section, we investigate supervised machine learning techniques that can be used to implement such an extension of our data set by labelling the steps present in the cells automatically.

## Extending DASWOW Using Supervised Machine-Learning

The workflow analysis presented in the previous section provided us with insights into the data science step a developer is engaged in. However, to derive further insights that can provide an important basis to both understand the data science workflow practices and provide appropriate tooling, large-scale analysis on an even larger data set of workflow information would be beneficial. As manual labelling does not scale to very large collections, in this section, we investigate to what extent DASWOW can be used to train a classifier capable of providing highly accurate data science step annotations. To that end, this section aims to answer ‘*How well do current supervised machine learning methods perform when predicting data science steps in Python Jupyter Notebooks?*’. First, we describe the preparation of the data, and then the methodology of developing the classifiers, followed by an evaluation of the classifiers’ performance. Subsequently, we provide an evaluation of the representativeness of the classifier results by comparing the workflow analysis based on manually annotated and predicted labels.

### Methodology

We first describe our data preparation process which includes feature engineering and data pre-processing.

#### Feature Engineering

In addition to the data science step labels for the training data, we generated 15 features (lexical/textual and numerical) to represent a code cell. They refer to the content of code cells, their metadata (e.g., cell number), and information related to their neighbouring cells (e.g., markdown_heading). Given markdown cells can provide useful information, for each code cell, we consider the first line (Pimentel et al. 2019) of the preceding markdown as one of the features. We also considered the usage of repository information (such as forks, watcher, stars, owner information etc.) of a notebook; however, given their sparsity and initial insight into their informativeness, we decided not to use them further. Moreover, using an external library Radon^7^, features such as number of distinct operators and operands, bugs, time and complexity, number of code cells in a notebook, number of markdown cells in a notebook, etc., were generated at the notebook level. However, we did not consider them for classification after an initial evaluation as the library requires the code to be represented as .py file, which results in the loss of its structural information. Instead, we generated code metrics relevant to each code cell using custom functions. The detailed list of features, its definitions and the rationale for its inclusion are listed in the Appendix (Section Appendix). The features are extracted directly from the explicit information in the notebook (e.g., execution count) or generated programmatically^8^ (e.g., number of variables). As a result, we have the following sets of features to represent each code cell:

*Cell features*: are based on the content available in the cell itself, such as lines of code, comments, execution count as provided by its Jupyter environment etc., its meta-data and related components (such as output obtained from its execution).
*External features*: indicate the man-page information about the libraries imported in a given cell which gives more information about the functions and use of a library.
*Software metrics*: contain code metrics (e.g., number of variables) for a given cell using custom functions we implemented.

In total, each code cell in DASWOW is represented by 15 features.

#### Data Preprocessing

Before applying the classification model, we apply common preprocessing procedures to the features: where applicable, we apply pandas.factorize() to obtain the numerical representation (by one-hot encoding) of non-numerical features with a distinct set of values (e.g., output_type). For the lexical features like code, comments, and markdown heading, we first remove the stop words by setting the language to ‘English’ and punctuation. We then use TfidfVectorizer^9^ to tokenize the lexical features by extracting uni-, bi-, and tri-grams. We also enable inverse-document-frequency re-weighting. Using a trial, we set the threshold for document frequency at a maximum of 0.2% and a minimum of 2% to ignore corpus-specific stop words and infrequent words, respectively. As a result, lines of code of a cell are represented by a feature vector of size 48,485. When using numerical features, we apply *standardisation*^10^ to the features. We use the standard *chi2* method for feature selection to select *k* most relevant code features (token) and discuss in detail the performance of classifiers over various sizes in Section 11.

#### Evaluating the Classification Methods

As each data point in our dataset contains one *primary label* and zero or more *secondary labels*, we investigate two relevant multi-class classification (more than two target classes) problems: single-label and multi-label^11^, ^12^. Single-label classification assigns one label whereas multi-label classification assigns one to many labels per data point. For the single-label classification, we use the *primary label* as the classification target and predict one label per code cell. For the multi-label classification, we use all the applicable labels i.e., *primary label* and *secondary labels* as targets and, as a result, predict multiple labels per code cell. In the case of multi-label classification, we use the binary relevance approach implemented via the OnevsRest strategy. We formally define the classification of data science steps as follows.

Given a dataset *D* containing a set of samples {*X*,*y*}, where *X*_*i*_ is a feature vector and *y*_*i*_ is its corresponding label with *y*_*i*_ ∈{1,...,*K*}, a set of classifiers *f*_*k*_(*x*|*𝜃*_*k*_) is learned, where *𝜃*_*k*_ represents its corresponding optimal parameters. In our classification problem, *K* = 10 (Table 1).

For an unlabelled sample *x*, single-label classification learns the following decision function:

1 $$ \hat{y} = \arg\max_{k \in \{1,\ldots,10\}} f_{k}(x) $$

For the multi-label classification learns the following decision function:

2 $$ \hat{y} = \{y_{1},y_{2},\ldots,y_{K}\} \text{, where } y_{k} = \left\{\begin{array}{ll} 1 & \text{if $f_{k}(x) >= t$}\\ 0 & \text{otherwise} \end{array}\right. \text{ for } k \in \{1,\ldots,10\} $$

For the classification task^13^, we applied random sampling on DASWOW to create the training (60%), validation (20%) and test (20%) sets (Lever et al. 2016). To retain all the data points of a given notebook in a single set and as a result, to train the classifier with (latent) order information relevant to a notebook, the random sampling was executed on *filenames* rather than data points (‘code cells’) themselves. As a result, the training, validation and test sets contain 282, 94, and 94 notebooks, respectively. The validation set is used in GridSearchCV^14^ for 10-fold cross-validation using scikit’s PredefinedSplit^15^. The predefined split allows us to retain all cells belonging to a notebook together and at the same time perform n-fold cross-validation. The test set is used to evaluate the performance of the classifier.

##### Classifiers

Given that the goal is to illustrate the performance that a classifier provides for data science predictions, we opted for comparing a set of well-known, off-the-shelf classifiers — an approach that can serve as a baseline for future explorations of supervised classifiers. Specifically, we investigated the performance of six classifiers: Random Forest (RF), Decision Tree (DT), Gradient Boosting (GB), Linear Support Vector (LSV), Support Vector (SV), and Logistic Regression (LOG). We use standard scikit-learn implementations for each of the classifiers.

In order to assess their usefulness, we create and evaluate the following six sets of feature combinations from the 15 features for each of the classifiers based on the (additional) information they provide while keeping *code* as the focal point:

1. *code* containing only the lines of code (represented by text in the DASWOW)
2. *code-comment* containing the lines of code and additional information from the comments (represented by comment in the DASWOW)
3. *code-stat* containing the lines of code and the additional software metric information (represented by linesofcomment, linesofcode, variable_count, function_count in the DASWOW) for a cell
4. *code-cell* containing the lines of code and other cell-related features
5. *all-features* containing all (code, comment, stat, and cell) the features.
6. *all-features-without-code* containing all the features excluding the lines of code.

##### Classifier evaluation metrics

To evaluate the performance of the classifiers, we consider the *F1-score*^16^ (Tsoumakas and Vlahavas 2007), which is the harmonic mean calculated using *precision* and *recall*:

3 $$ f1=2 \cdot \frac{precision \cdot recall}{precision+recall} $$

whereas *precision* and *recall* are derived from true positives (TP), true negatives (TN), and false positives (FP) as below:

4 $$ precision=\frac{TP}{TP+FP} $$

5 $$ recall=\frac{TP}{TP+FN} $$

We also look at other most common evaluation metrics like accuracy^17^ for single-label classification, and subset accuracy, hamming loss,^18^ and jaccard similarity^19^ for multi-label classification (Park and Read 2018). Note that in multi-label classification, instead of accuracy, subset-accuracy (also called ‘Exact-Match’ accuracy), which indicates the percentage of samples that have all their labels classified correctly, is calculated. The Exact-Match accuracy is considered a harsh metric as it requires all of the labels for a given sample to be correctly predicted (i.e., if one of the labels in the set does not match, it is considered incorrect).

### Results

We first provide a comparison of the performance of various classifiers for both single and multi-label classification. Then, we proceed to discuss the best-performing classifier in detail. We performed a detailed feature ablation study that takes into account both the combination of features and the code feature vector size over different classifiers, which we later discuss in this paper (refer to Section 5.2.3).

#### Predicting one Label per Code Cell

The accuracy and F1-score of all the classifiers evaluated over a different set of features are listed in Table 7. The size of the code feature vector is 1000 which is the top performing amongst all.

**Table 7:**
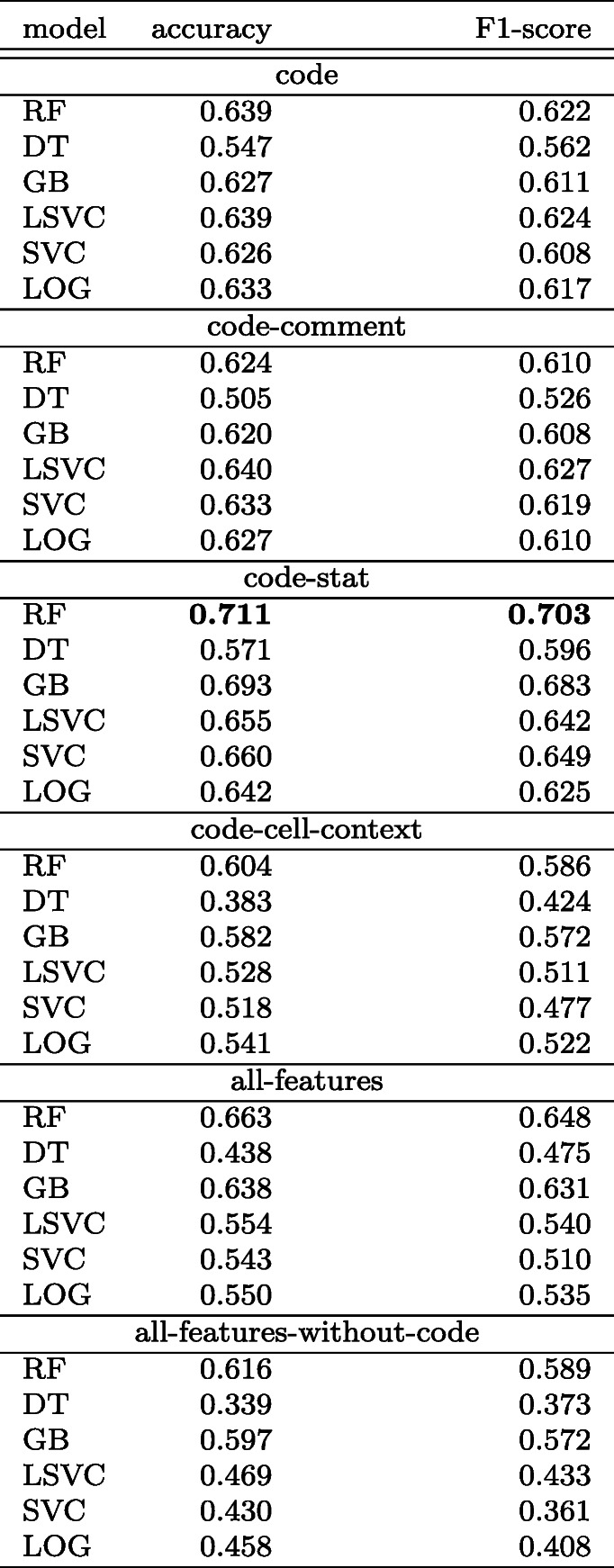
Single-label classification: Comparison of accuracy and F1-score metric of classifiers over a different set of features

The RF classifier trained with *code-stat* features has the highest accuracy of 71.1%, closely followed (69.3%) by the GB classifier trained with *all-features*.

##### Best performing classifier

We take the best performing classifier (RF) trained with *code-stat* for further analysis using GridSearchCV. The best performing parameters evaluated by GridSearchCV (with parameters n_estimators= 90, class_weight=’balanced_subsample’, random_state= 500, criterion=’gini’) achieves a *weighted average F1-score* of 0.698.

**Fig. 9:**
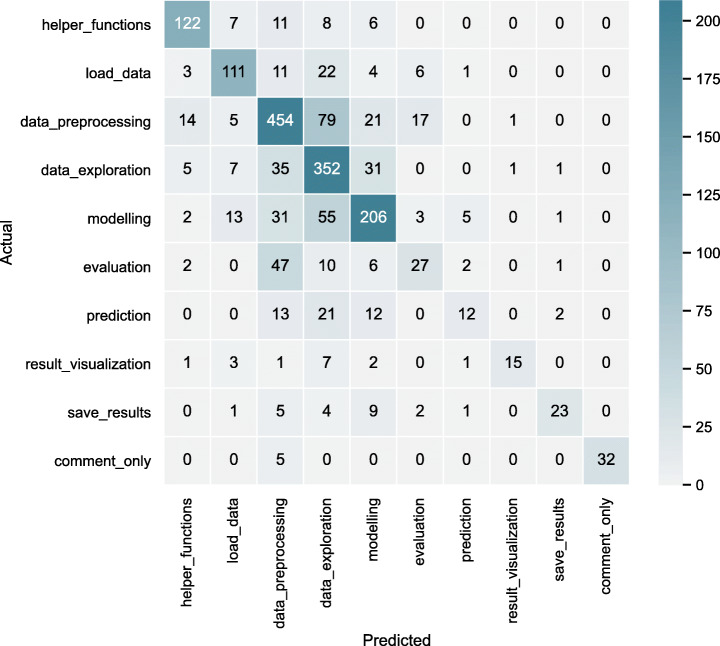
Random forest: single-label classification confusion matrix

Table 8 shows the classification_report for the RF classifier.

data_preprocessing has a precision value of 0.74 and a recall value of 0.77. The reason for such a result could be that the lines of code that performs data_preprocessing do not have many unique structures (e.g., plt.figure() in the case of result_visualization or DecisionTreeRegressor() in the case of modelling). In the case of data_exploration, even though it has a lower precision score, the recall score is higher indicating that many of the labels are predicted as data_exploration even when they are not; the confusion matrix visualises this behaviour (Fig. 9).

**Table 8:** Single-label classification: performance metrics of RF classifier

	single-label classification:			
	best performing GB classifier			
labels	precision	recall	F1-score	support
helper_functions	0.82	0.79	0.81	154
load_data	0.76	0.70	0.73	158
data_preprocessing	0.74	0.77	0.75	591
data_exploration	0.63	0.81	0.71	432
modelling	0.69	0.65	0.67	316
evaluation	0.49	0.28	0.36	95
prediction	0.55	0.20	0.29	60
result_visualization	0.88	0.50	0.64	30
save_results	0.82	0.51	0.63	45
comment_only	1.00	0.86	0.93	37
accuracy			0.71	1918
macro avg	0.74	0.61	0.65	1918
weighted avg	**0.71**	**0.71**	**0.70**	1918

evaluation and prediction have lower recall values (< 0.30) and precision (<= 0.55) values. We suspect this is because both these labels occur usually in conjunction with modelling (as found in our analysis (refer to Section 4.1)). Also, this label information is lost as we have used only *primary label* here. It is also possible that the presence of features relevant to other labels in a given data point might be a source of confusion for the classifier. For example, consider a code cell performing modelling as a main activity (captured by *primary label*) that also performs evaluation in the later lines of code (captured by *secondary labels*). In this case, the single-label classifier will encounter features that might be relevant to both modelling and evaluation.

#### Predicting Multiple Labels per Code Cell

We compare the performance of classifiers over various sets of features for multi-label classification. The results are listed in Table 9. The size of the code feature vector is 2000 which is the top performing amongst all.

**Table 9:**
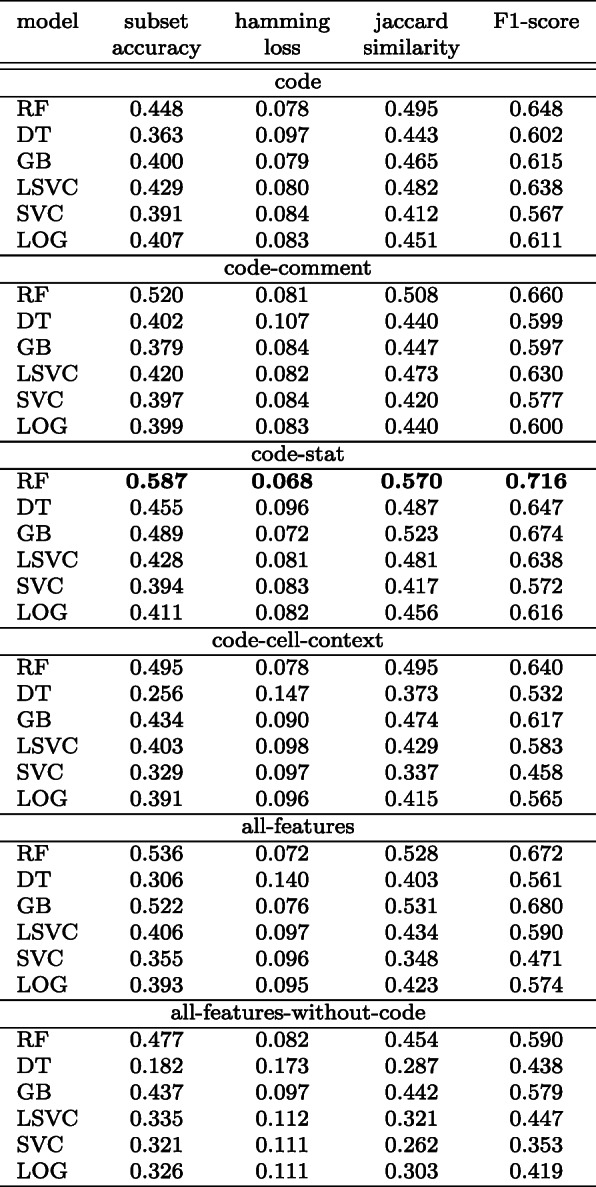
Comparison of a set of metrics of classifiers over a different set of features: multi-label classification

The RF (random forest) classifier trained with *code-stat* achieved the highest *F1-score* of 0.716 and a subset accuracy of 0.587. Since we use a total of ten classification labels in multi-label classification, we expected the subset accuracy to be less as it is a harsh metric. Table 9 shows that all of the classifiers have a hamming loss that is < 0.180. A low hamming loss score means that the proportion of labels predicted incorrectly is low. Jaccard similarity, which measures the similarity between the set of predicted labels and the corresponding set of true labels, is a midpoint between hamming loss and subset accuracy (Park and Read 2018) and is more likely to show a balanced view of the classifier’s performance. The RF classifier has a Jaccard similarity coefficient score of 0.570 i.e., the set of labels predicted is 57% similar to the set of true labels. The GB classifier trained with *code-stat* is the second-best performing with a Jaccard similarity score of 0.489 and an F1-score of 0.674.

##### Best performing classifier

We take the best performing classifier (RF) trained with *all-features* for further analysis using GridSearchCV. The best performing parameters evaluated by GridSearchCV is Random Forest (with parameters n_estimators= 100, class_weight=’balanced_subsample’, random_state= 500, criterion=’gini’).

**Fig. 10:**
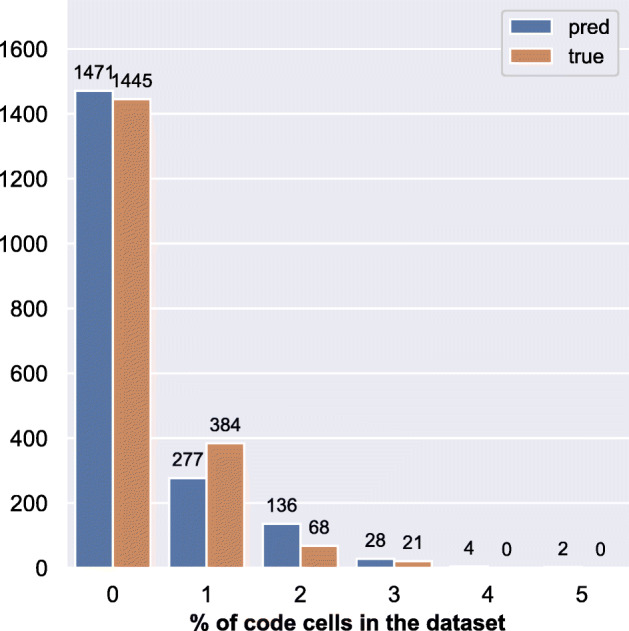
True vs predicted label set composition

Table 10 shows the classification_report for the RF classifier. Most of the classification labels have precision value >= 0.74 except data_preprocessing (0.69) and result_visualization (0.43). In terms of recall, helper_functions, data_preprocessing, data_exploration, and comment_only have recall values equal to or greater than 0.75. The recall value for result_visualization has worsened more than double compared to the single-label classification indicating an increase of false negatives. On the other hand, for result_visualization, the precision value has worsened along with the recall value indicating the classifier performs poorly with this label. While maintaining the weighted average of *F1-score*, multi-label classification has improved in average *precision* by 9.9*%* whereas degraded in *recall* by 5.6*%*. We also notice that the RF classifier did not assign any label to 6.4% of the data points (see Fig. 10 showing the comparison of the distribution of labels in the true vs. predicted set).

**Table 10:** Multi-label classification: performance metrics of RF classifier

	multi-label classification:			
	best performing RF classifier			
labels	precision	recall	F1-score	support
helper_functions	0.91	0.82	0.86	286
load_data	0.88	0.66	0.75	191
data_preprocessing	0.69	0.75	0.72	437
data_exploration	0.80	0.79	0.79	801
modelling	0.78	0.58	0.67	361
evaluation	0.74	0.30	0.43	171
prediction	0.77	0.33	0.47	90
result_visualization	0.43	0.16	0.24	97
save_results	0.84	0.53	0.65	30
comment_only	1.00	0.86	0.93	37
micro avg	0.78	0.67	0.72	2501
macro avg	0.78	0.58	0.65	2501
weighted avg	**0.78**	**0.67**	**0.71**	2501
samples avg	0.74	0.71	0.71	2501

While this is a concern which is reflected in a low *weighted average recall* score, the classifier has a higher *weighted average precision* i.e., not many labels are inaccurately assigned.

#### Feature Ablation

##### RQ-2a Do non-code artefacts like markdown or comments and code metrics improve classification accuracy?

For multi-label classification, the performance of the set of features follows the pattern:

*F*1(*all*-*features*-*without*-*code*) < *F*1(*code*-*extend*) < *F*1(*all*-*features*) <= *F*1(*code*-*comment*) <= *F*1(*code*) < *F*1(*code*-*stat*)

Hence, the answer to **RQ-2a** is that code features along with statistical information perform better in the classification of data science steps.

##### RQ-2b Does the feature set size have any effect on classification accuracy?

Apart from investigating the effect of six sets of feature combinations: *code*, *code-comment*, *code-stat*, *code-cell*, *all-features*, and *all-features-without-code* on the classifier performance, we also looked into the effect of the size of the code feature (i.e., by selecting *k* tokens using *chi2*) through an ablation study to answer our research questions.

*Results:* Table 11 shows the performance over different sizes of feature vector. Based on the classifiers evaluated, we noticed that there is no clear pattern in the performance of the classifiers depending on the size of the code feature vector. While a code vector of size 5000 or 10000 performs better, increasing the size beyond 1000 does not seem to promise significantly better performance. As a result, the answer to **RQ-2b** is unclear. However, we found that the classifier trained with 470 notebooks (a larger set of data points) performs better than a subset of randomly selected 100 notebooks, which had an F1-score of 0.48.

**Table 11:**
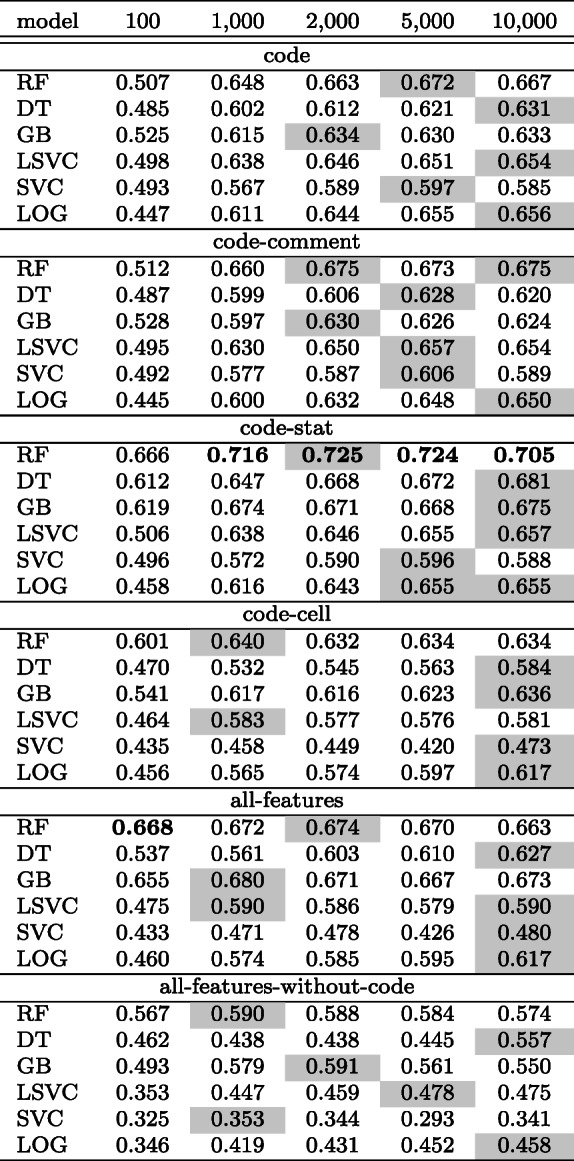
Multi-label classification: Comparison of *F1-score* over different set of features and sizes of the feature vector

Bold numbers indicate column-wise top performers and numbers in coloured background cells indicate row-wise top performers

#### Summary

The evaluation of classifiers in Section 5.2 showed the capability of existing supervised methods in extending DASWOW for large-scale workflow analysis on data science notebooks. Even though the classifier does not assign any label to 7% of the data points, the frequency distribution and transition probabilities of the predicted set show the effectiveness of the classifier. For a classifier to effectively extend the DASWOW dataset, it should produce zero false positives. False positives compromise the quality of the resulting dataset and, therefore, are costly. As an inaccurately labelled data point is worse than an unlabelled data point, this, in our case, can lead to an inaccurate workflow analysis. For this reason, a classifier that has a good *F1-score* and at the same time a better precision score is useful. In our evaluation of classifiers, multi-label classification with RF classifier has a *weighted average F1-score* of 0.71 and a *weighted average precision* of 0.78 compared to single-label classification with RF classifier’s *weighted average F1-score* of 0.70 and a weighted average precision of 0.71. Hence, in this case, multi-label classification is preferable.

### Workflow Analysis-Based Evaluation

In this section, we evaluate the results of the classifier in terms of the workflow analysis. We apply the same methodology followed in Section 4 to the dataset containing the data science step labels predicted by the best performing multi-label RF classifier (see Section 5.2) and compare the workflow analysis results obtained in both cases (i.e., a dataset with manually obtained labels and dataset with predicted labels) in order to assess the potential of the supervised classification methods in extending the dataset to support large-scale analysis. We present these results for each of the three individual research questions:

#### Frequency (RQ-1a)

**Fig. 11:**
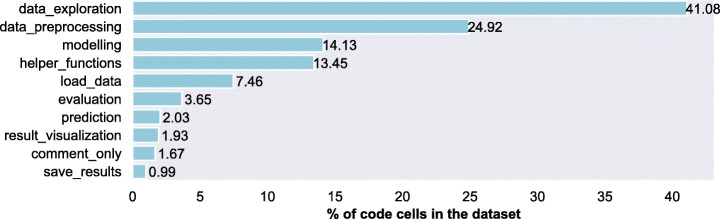
Number of cells per label: all labels

We found that the distribution of the labels in our prediction set (see Fig. 11) is close to the distributions obtained using the training dataset except for a few labels. Particularly, there is a reduction in result_visualization. We computed Kendall’s rank correlation (*tau*) and found that the samples from training and the predicted set of labels are highly correlated with a coefficient of 0.956. The result shows the effectiveness of the classifier.

Furthermore, the distribution of no of labels per cell in the predicted set has similar composition to the true composition, with around 76% of the cells in our data set having one data science step, 14% having two steps, and 2% having more than two steps. However, around 7% of the cells are unassigned, resulting in Kendall’s rank correlation co-efficient of 0.60 compared to the composition in DASWOW.

#### Transitions (RQ-1b)

**Fig. 12:**
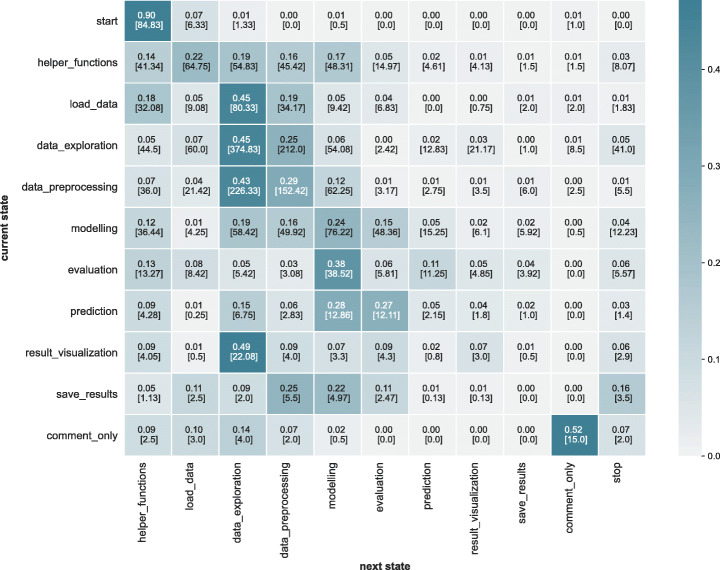
Transition matrix probabilities across data science workflow - predicted

Transition probabilities for the predicted set of labels based on the equal strategy (refer to Section 4.2) are shown in Fig. 12.

Given a current state in the transition, we compare the probabilities to the next state between the predicted set and DASWOW using Kendall’s tau. Hence, we essentially compare each row of Fig. 5 with the respective row of Fig. 12 to compute the correlation. Table 12 shows the correlation results. We found a statistically significant correlation (*p* <= 0.05) between the state transition probabilities in these figures, which shows the effectiveness of the classifier in predicting the labels resulting in comparable transition sequences.

**Table 12:** Kendall’s correlation coefficient of transition probabilities to the next state between the training and predicted set given a current state in the data science workflow

current_state	Kendall’s tau
start	0.861
helper_functions	0.881
load_data	0.697
data_exploration	0.891
data_preprocessing	0.709
modelling	0.673
evaluation	0.709
prediction	0.673
result_visualization	0.661
save_results	0.611
comment_only	0.740

#### Summary

Our workflow analysis (Section 5.3) on the predictions from classifier reveals similar insights to Section 4 on individual data science steps. We believe that the performance of the classifier can be enhanced by other approaches, including interactive methods like active learning leading to a more accurate data science workflow analysis. Such an analysis would reveal patterns and provide insights that support data science development and automation of data science workflows.

## Discussion and Implications

In this section, we further discuss the insights revealed through our workflow analysis on data science code using DASWOW and highlight its implications along two dimensions: the development and maintenance of data science workflows. We also identify and categorise the use cases where the data science step annotations can be of value.

### Better tool support for computational notebook IDEs

Data scientists lack proper coding assistance within computational notebooks (Chattopadhyay et al. 2020; Olabarriaga et al. 2014). Features that already exist in traditional IDEs should be extended to computational notebooks like Jupyter as “data scientists are akin to software developers, but constrained to a very specialised environment” (Olabarriaga et al. 2014). Here, data science annotations can act as task tags or provide metadata information in IDE tools. For example, they can support type hierarchy (Robillard et al. 2004) by grouping data science procedures since data scientists currently cannot “explore the API and functions” in the notebook (Chattopadhyay et al. 2020). They can help data scientists in (re-)structuring a notebook, one of the pain points in notebook development (Head et al. 2019; Titov et al. 2022; Dong et al. 2021), by identifying the activities they perform and splitting and(or) merging the cells.

### Supporting exploratory programming

Data scientists spend a large fraction of cells (as a result, time and effort) exploring and pre-processing data (refer to Section 4.1). While the effort required for pre-processing tasks has already received attention for automation support, the exploration itself has been traditionally considered a manual task left to the data scientists and domain experts. There is still room for creating tools that automate data exploration in order to assist data scientists. For example, a tool that provides a sample set of exploration paths (“garden of forking paths”) (Gelman and Loken 2013; Schweinsberg et al. 2021) to be tested by the user would be useful.

At times, data scientists implement more than one data science step per cell (refer to Section 4.1). Our results provide additional empirical evidence to the finding that notebooks are usually ’messy’ and lack logical structuring (Head et al. 2019; Kery et al. 2019; Rule et al. 2018) due to the exploratory nature of data science coding. With the help of data science annotation, tools could support the automatic modularisation of a code block by combining or merging cells within the notebook.

Furthermore, data scientists transition within and between data science steps iteratively throughout a data science workflow (refer to Section 4.2). Together with a lack of structure, this can lead to navigation and comprehension difficulties for data scientists trying to understand others’ or their own notebooks and further hinder collaboration (Head et al. 2019; Kery et al. 2019; Chattopadhyay et al. 2020). We also observe in the transition patterns that, many times, a cell performing a certain activity is followed by another cell that performs the same activity. This behaviour is not intuitive. For instance, a cell performing data exploration activity is followed by another cell which performs the same activity 43% of the time. This finding calls for larger and more specific support to data scientists in the context of workflow design, development, and management. However, to the best of our knowledge, current workflow automation systems do not take into account this iterative pattern of steps in a workflow (Heffetz et al. 2020; Drori et al. 2021).

### Automating data science coding

Chattopadhyay et al. (2020) identify lack of support for coding (e.g. live templates, programming examples) as one of the pain points for data scientists. As a result, data scientists spend a lot of time browsing examples on the internet (e.g., Stack Overflow) during their development (Chattopadhyay et al. 2020). By integrating a tool or a plugin that dynamically classifies the code being written according to the data science step, a programming environment can suggest relevant coding templates. Particularly, such a support in modelling and data_exploration steps may be helpful as they are frequently cloned (refer to Section 4.4) and may cause code dependency problems (Kery et al. 2018) in the notebook. Such a tool may also learn from the user’s acceptance of suggested annotations to improve itself and customise its future recommendations. They can also provide complementary support to existing recommender systems (Watson et al. 2019; Svyatkovskiy et al. 2019) (that focus on certain steps) by automatically identifying and predicting the current (or next) data science activity so that relevant examples and alternatives (e.g., a template recommender tool may use the identified step in order to provide appropriate examples) are suggested to the data scientist.

Our workflow analysis also reveals that data scientists invoke or import many helper functions, including external libraries, while performing a data science task. Identifying the right libraries to use and managing their versions (Wang et al. 2021b; Chattopadhyay et al. 2020) can become time-consuming and inconvenient for data scientists over time. By predicting the next data science step, an automatic library recommender and manager with an in-built function of adding the code can then recommend relevant libraries.

### Support in navigation and comprehension of data science notebooks

Our empirical findings show several potential issues that may give rise to navigation difficulties, as also reported by other interview-based studies (Chattopadhyay et al. 2020; Head et al. 2019; Kery et al. 2019). Implementing multiple steps that require logical separation within the same cell (Head et al. 2019), multiple iterations (Chattopadhyay et al. 2020), lack of an overview of the workflow — data science steps and the transitions between them due to the notebook’s linear structure, could all contribute to a messy notebook that is difficult to navigate through and comprehend. In such cases, data science step annotations can act similar to pre-defined annotations in traditional IDEs. Together with a code browser, they can allow the users to navigate, access, and debug the relevant block of code easily (Storey et al. 2009; McCormick and De Volder 2004).

### Support in workflow management

Data science annotations can support transformation and integration of data science notebooks as workflows into other scientific workflow systems like Taverna and Wings (Garijo et al. 2013a). Furthermore, they can enable the analysis of the evolution of the data science workflows and enable users to find, interpret, modify, and reuse relevant (sub-)workflows.

## Limitations and Threats to Validity

One limitation in our study is that our annotation process during the dataset generation relied on few annotators. However, we selected the annotators after a pilot study in order to generate a dataset with consistent and high-quality annotations. Further, given expert annotations are expensive and hard to obtain, improvements to the dataset can be introduced in future using techniques like active learning. In addition to the disagreements we discussed in Section 3.3, our annotation task also revealed the difficulties that arise in understanding a notebook (Rule et al. 2018; Pimentel et al. 2019). For example, a cell may contain a function definition that is executed only later in the notebook. In such cases, we refer to other steps that may be present in the cell as a primary task instead of the function definition. Another example of ambiguity arises if a cell contains code that performs several activities. Here, deciding a single *primary_label* is difficult. While annotating each line of code may be a solution, this is, however, tedious for annotators and also expensive. We address this currently by preferring the first appearing step as *primary_label* and marking the others as *secondary_labels*. We see this as a good compromise given their low frequency of appearance (refer to Table 6).

Another limitation is that the supervised classification method does not perform well on certain labels. For example, evaluation, prediction, and result_visualization labels had a comparatively low F1-score. This is due to the unbalanced nature of the dataset, i.e., the rare appearance of these labels in the dataset. From our analysis, we view this as an inherent disadvantage that should be tackled with upsampling. In the case of result_visualization, we suspect another reason for the low classifier performance is that the code structure is similar to data_preprocessing and data_exploration. We believe this could be improved by considering the sequential information around the label and is left for future work.

Validity concerns arise primarily from generalising the results of a set of notebooks that are taken from GitHub. To mitigate this issue, we applied random sampling to select the notebooks from the widely used corpus by Rule et al. (2018) to create DASWOW. Also, the descriptive statistics on DASWOW corroborates with findings on the entire corpus. However, we cannot rule out that the results from workflow analysis of notebooks from other platforms like Kaggle, where data scientists compete with peers and write well-documented notebooks (Gil et al. 2010), may diverge.

Another possible concern could be the representativeness of the sample analysed in this work. To address this issue and also to enable further validation, we relied on the widely used notebooks dataset created by Rule et al. (2018) as the source. We selected the notebooks through random sampling and through then manually verified the purpose of each of the notebooks before considering them for our analyses.

## Conclusion

Understanding the way data science is designed, implemented and reported by data scientists is important to provide tools and frameworks that enable high-quality data science. In this paper, we presented an empirical analysis of the data science workflows in real-world practical implementations. To that end, we first provide an expert-annotated dataset (DASWOW) for workflow analyses. Our analysis confirms previous preliminary evidence that most real-world notebooks have iterative nature and reveal unique patterns in the data science workflow.

Through an evaluation of existing classification methods, we also showcased that DASWOW can help in extending the dataset via machine learning methods. To that end, we evaluated a set of classifiers for several sets of features in order to understand their relevance in the classifier performance. We also extended our workflow analysis on the predicted set of labels. Our results indicate that such an extension via machine learning methods is indeed possible and provide a baseline for future improvements (e.g., via more sophisticated feature construction or deep learning techniques).

The results of our workflow analysis also provide valuable insights and motivation for developing tools that support, guide, and automate data science coding. Among other things, they raise questions about how to support the clearly iterative nature of data science explorations and the need for further understanding of the intermingling of various tasks within cells. As such, we hope that data science and the insights provided by our analyses will help (1) build better tools for supporting data scientists’ analyses and (2) bootstrap further analyses of other data science notebook collections.

## Data Availability

The datasets generated and analysed during the current study are available as a part of our replication package at the following link: 10.5281/zenodo.5635475

## Appendix: A

### A.1 Instructions for Ground Truth Annotators

The main goal of this *task* is to design and implement a method that automatically classifies different parts of a Data Science notebook, so as to label the steps of the Data Science process that are present in the notebook. In order to train and evaluate the method, we need to curate a ground truth. Therefore, your input as an expert labeller is very much appreciated.

You will be given a set of notebooks to be labelled, a set of possible labels and your task is to indicate for each cell of the notebook one or more relevant label(s), that describe the purpose of the code appearing in the cell. We would also like you to indicate how confident you feel about your answers. Below, you will find detailed information about the meaning of the labels and things to take into account.

In this annotation task, you will need to consider the following files for each notebook to be annotated:

- *notebook_name.ipynb*: the Python notebook to be analysed and labelled.
- *notebook_name.ipynb.xlsm*: a macro-enabled excel worksheet, where you should annotate the notebook.

#### A.1.1 Instructions

Please read the instructions before proceeding with the labelling task.

##### How to annotate one notebook

- Open the Python notebook and the annotation file in any compatible software (e.g., use Jupyter for the notebook and Excel for the annotation file).
- Read the set of possible labels and their meaning (see also Section Appendix).
- For each cell in the notebook, please read the content and consider the line(s) of code, plots, visualizations and comments. Please ignore markdown.
- Decide the purpose of the cell, and assign one or more of the available labels. In the annotation file (*notebook_name.ipynb.xlsm*), please mark with *1* the label(s) you would like to assign to the cell.
- Also, determine the primary data science process performed in the cell. Record the observation by choosing the appropriate label in the primary_label column in the annotation file.
- If, for a given cell, you do not agree with any of the given labels, please indicate the label that you think is relevant in the *other_label* column.
- You have the option of adding some notes in the *notes* column, if you would like to enter any observation about the cell (e. g. if it was hard to decide the label or anything you think it is important to be mentioned). You do not need to enter notes for every single cell.
- Once you have completed the labelling of all cells, please indicate in the last two rows of annotation file the following information:
  1. confidence percentage: how confident you felt when providing the labels (with a value between 0 (not confident at all) and 100 (very confident)).
  2. notebook score: give a general score for the notebook, based on the clarity and understandability of the code (with a value between 0 (not clear at all) and 100 (very clear)).

##### General tips

- The annotation should be done based on the available implementation in the notebook. Please do not execute the code of the notebook to decide the label.
- Note that sometimes, it is useful to go through the whole notebook to be aware of the context of the cells.

#### A.1.2 Classification labels

We defined a set of 9 labels: 3 of them are labels that refer to general coding actions, and 7 labels correspond to the main steps in the data science process.

**General labels**

##### load_data

This label indicates that the cell is just loading data into the Jupyter notebook environment. The code can be for loading one or multiple data sets of any type (e.g., .csv, .pkl, .jpg, .png, .hdf5).

##### helper_functions

This label should be used when the cell contains code that supports the execution of the code in the notebook. These helper functions can be *import statements* (e.g., import pandas as pd) or other piece of code that is not directly related to the data science activity at hand and rather are useful functions in scripting. For example, built-in Jupyter notebook magic commands. A concrete example would be: *%matplotlib inline*, which sets the inline backend so that the output plots are displayed directly below the code cell that executes it. Some other examples include from IPython.display import Audio, and %pprint.

##### comment_only

If the cell contains only commented text, you should use this label. This label should not be used for markdown; please remember that you should ignore markdown.

**Labels for each of the data science steps**

##### data_preprocessing

Data preprocessing includes tasks such as cleaning, instance selection, data normalization, data transformation, feature extraction and feature selection. Data preprocessing ensures that the data does not contain irrelevant, redundant and inconsistent data. 20

##### data_exploration

Data exploration^21^ or exploratory data analysis is an approach to initial data analysis, where a data scientist inspects the content of a dataset in order to understand the nature and characteristics of the data. This step, that often contributes to the identification of patterns in the data, is helpful to identify research hypotheses and decide on the modelling. Data exploration often involves visual exploration of the data.

##### modelling

Modelling is the process of applying (or fitting) statistical models and algorithms to the data in order to “perform a specific task without explicit restrictions, relying on models and inferences instead”^22^. In simple terms, training a classifier or a neural network to learn from the data is modelling.

##### prediction

Prediction is an important step as many of the data science tasks are predictive modelling tasks. This label refers to the generation of an outcome that was previously unknown, by applying the model built to new data.

##### evaluation

Evaluation refers to the process of evaluating the model using evaluation metrics like accuracy, or F1-score. In the context of model selection, the evaluation step can be used to compare different models. Evaluation is also done to compare different models 23.

##### result_visualization

Result visualization is the graphical representation of results (via elements like plots, tables and other graphs). Please note that we differentiate between data exploration and result visualization. The purpose of the former is to better understand the data, while the latter focuses on visualizing e. g. a tested hypothesis, or a performance comparison.

##### save_results

This label should be used when the cell is primarily creating a persistent copy of some results (e. g. serializing the content of a variable into a CSV file).

### A.2 List of features

Table 13 lists all the features^24^ extracted to form the DASWOW.

**Table 13:** Data science code features for classification

Feature	Description	Rationale for inclusion
**Cell features** are extracted from the content of individual cells, their related components (such as the output obtained from the cell execution), and information about neighbouring cells.		
*filename‡*	Filename indicates the name of the notebook file where the cell comes from.	It provides metadata information about the cell by providing information about where it resides. Cells containing the same filename but with a different cell number provide positional information about the cell that may indicate the presence of certain data science steps.
*cell_number*	Cell number indicates the position of the cell in a given notebook. Cell number along with filename uniquely identifies a cell in the dataset.	This information about a code cell may be indicative of the type of the data science action in the cell as different data science steps are expected to occur at a certain phase in the cycle (Wang et al. 2019a; Zhang et al. 2020a; Aggarwal et al. 2019; Muller et al. 2019b; Souza et al. 2020). For e.g.,, data_preparation and data_exploration generally occurs in the initial stages of data science workflow whereas modelling and evaluation at a later stage.
*execution_count*	Execution count indicates the order in which a cell was executed by the user in a notebook.	It provides execution-level information about a code cell which could be indicative of a certain data science step. For example, a lower number may suggest a step at the beginning of the data science workflow.
*text*	Text contains the content of the cell, which includes either the code text or markdown text depending on the cell_type (markdown or actual executable code).	Different blocks of code implement different data science actions and hence can exhibit patterns that identify the data science step.
*comment*	All the comments in a given code cell. This feature is valid only for code cells.	It provides (additional) information about the intended purpose of the code present in a particular cell (Rule et al. 2018).
*output_text*	This indicates the content of the output. For example, image/png or text/plain. This feature is valid for the cells of cell_type output.	The output information of a given code cell may be indicative of a certain data science step. For example, image/png may indicate a data_exploration step since data scientists use visualisation techniques to explore data.
*output_type*	This indicates the type of the output. For example, display_data. This feature is valid for the cells of cell_type output.	It provides information about the type of the output (stream data or rich mime-type output) that could be indicative of a certain data science step.
*code_line_before*	code_line_before indicates the last line of code in a code cell preceding the current cell. In the case of the cells of cell_type markdown or raw_nb, this information is obtained from the code cell preceding it (may or may not be immediately preceding).	The previous code cell’s line of code provides additional context by providing information regarding the surrounding structure of a particular data science step (Bacchelli et al. 2012).
*code_line_after*	code_line_after indicates the first line of code in a code cell following the current cell. In the case of the cells of cell_type markdown or raw_nb, this information is obtained from the code cell following the current cell (may or may not be immediately succeeding).	The following code cell’s line of code provides additional contextual information and may provide indications about the previous step.
*markdown_heading*	markdown_heading indicates the first line of a markdown cell. In the case of the cells of cell_type code, this information is obtained from the markdown cell preceding it (may or may not be immediately preceding).	They are descriptive of the steps in the code cells that follow because data scientists tend to organise their code in sections for readability purposes ((Rule et al. 2018; Bacchelli et al. 2012)).
**External features** containing the detailed textual description of the imported libraries into a cell are included in order to extract information about its purpose.		
*packages_info*	This feature indicates the man-page information about the libraries imported in a given cell. The information contains the description of libraries as given in an external source: The Python Package Index (pypi)^a^.	It provides information about the libraries imported in the cell using a detailed description, thus providing a larger bag of distinctive words.
**Statistical/Metric features**: Statistical or Software Metric-based features are features that contain code metrics for a given cell. Below custom metrics are generated using class:NotebookMetrics and class:CodeCellMetrics. The metric-based features are included under the view that structural information of the notebook can provide information about the data science step.		
*no_linesofcode*	Lines of code indicates the total number of lines in a given cell of cell_type code.	It provides statistical information about the density of a code cell which could be indicative of the data science step because certain activities require a lot of lines of code than others. For example, data_preprocessing may have a lot of lines of code.
*no_linesofcomment*	Lines of comment indicates the total number of comment lines in a given cell of cell_type code. Any line that starts with ’#’ is considered a comment line.	It provides statistical information about the density of comments in a code cell which could be indicative of a certain data science step. For example, data_exploration may have a lot of lines of comments explaining the insights.
*function_count*	Function count indicates the number of functions in a given cell of cell_type code. Any line that starts with the keyword ’def’ is considered to be a function.	It provides statistical information about the number of functions created in a code cell which can indicate certain data science step (Barstad et al. 2014). For example, helper_functions will not have any functions defined.
*variable_count*	Variable count indicates the number of variables in a given cell of cell_type code. A variable is considered to be any keyword composed of alphanumerics and _ with a ’=’ to the right. Parameters are not considered.	It provides statistical information about cell structure and thus may help in identifying the data science step it is associated with (Barstad et al. 2014). For example, data_preprocessing may have a lot of variables compared to helper_functions.

^a^ https://pypi.org/

## Footnotes

1 to the best of our knowledge

2 The first author of this paper randomly sampled a set of notebooks and identified the purpose of the task implemented in them by manual inspection to select a set of 500 notebooks

3 We consider someone to be an expert when they have a minimum of two years of professional experience (in academia or industry) in implementing data science processes.

4 ^*^ indicates first author of this paper, # indicate experts from our lab, and ‡indicate freelance data scientists hired through Upwork.

5 potentially due to lack of comments

6 This includes type-2 clones that have identical syntax but different user-defined identifiers, type-3 clones which are modified clones with added or deleted or modified statements, and type-4 clones that have different syntax performing the same computation (Koenzen et al. 2020)

7 Radon introduction to Code Metrics - https://radon.readthedocs.io/en/latest/intro.html. Available at https://pypi.org/project/radon/.

8 To generate the features programmatically, we implemented a parser to parse the notebook and its metadata.

9 https://scikit-learn.org/stable/modules/generated/sklearn.feature_extraction.text.TfidfVectorizer.html

10 https://scikit-learn.org/stable/modules/generated/sklearn.preprocessing.MinMaxScaler.html

11 using scikit’s default OnevsRest

12 https://scikit-learn.org/stable/modules/generated/sklearn.Multi-class.OneVsRestClassifier.html

13 For feature ablation study, we utilise the validation set as training samples as well.

14 GridSearchCV: https://scikit-learn.org/stable/modules/generated/sklearn.model_selection.GridSearchCV.html

15 PredefinedSplit: http://scikit-learn.org/stable/modules/generated/sklearn.model_selection.PredefinedSplit.html

16 Scikit’s F1-score implementation: https://scikit-learn.org/stable/modules/generated/sklearn.metrics.f1_score.html

17 https://scikit-learn.org/stable/modules/generated/sklearn.metrics.accuracy_score.html

18 https://scikit-learn.org/stable/modules/generated/sklearn.metrics.hamming_loss.html

19 https://scikit-learn.org/stable/modules/generated/sklearn.metrics.jaccard_similarity_score.html

## Code Availability

All data and materials that support the findings of this study are available in our replication package at the following link: 10.5281/zenodo.5635475
